# *Andrographis paniculata* (Burm. f.) Wall. ex Nees: An Updated Review of Phytochemistry, Antimicrobial Pharmacology, and Clinical Safety and Efficacy

**DOI:** 10.3390/life11040348

**Published:** 2021-04-16

**Authors:** Sanower Hossain, Zannat Urbi, Hidayah Karuniawati, Ramisa Binti Mohiuddin, Ahmed Moh Qrimida, Akrm Mohamed Masaud Allzrag, Long Chiau Ming, Ester Pagano, Raffaele Capasso

**Affiliations:** 1 Department of Biomedical Science, Kulliyyah of Allied Health Sciences, International Islamic University Malaysia, Kuantan 25200, Pahang, Malaysia; 2 Department of Industrial Biotechnology, Faculty of Industrial Sciences & Technology, Universiti Malaysia Pahang, Kuantan 26300, Pahang, Malaysia; urbi.zannat@gmail.com; 3 Department of Pharmacology and Clinical Pharmacy, Faculty of Pharmacy, Universitas Muhammadiyah Surakarta, Surakarta 57102, Indonesia; hk170@ums.ac.id; 4 Department of Pharmacy, Faculty of Life Science, Mawlana Bhashani Science and Technology University, Santosh 1902, Tangail, Bangladesh; ramisa0799@gmail.com; 5 Department of Agriculture, Higher Institute of Overall Occupations-Sooq Al Khamees Imsahil, Tripoli 1300, Libya; ahmedtripoli87@gmail.com (A.M.Q.); akrmalazreq@gmail.com (A.M.M.A.); 6 PAP Rashidah Sa’adatul Bolkiah Institute of Health Sciences, Universiti Brunei Darussalam, Jalan Tungku Link, Gadong BE1410, Brunei; long.ming@ubd.edu.bn; 7 Department of Pharmacy, University of Naples Federico II, 80131 Naples, Italy; ester.pagano@unina.it; 8 Department of Agricultural Sciences, University of Naples Federico II, 80055 Portici, Italy

**Keywords:** antimicrobial agent, clinical trial, ethnopharmacology, infectious disease, medicinal plant, metabolites, natural products

## Abstract

Infectious disease (ID) is one of the top-most serious threats to human health globally, further aggravated by antimicrobial resistance and lack of novel immunization options. *Andrographis paniculata* (Burm. f.) Wall. ex Nees and its metabolites have been long used to treat IDs. Andrographolide, derived from *A. paniculata,* can inhibit invasive microbes virulence factors and regulate the host immunity. Controlled clinical trials revealed that *A. paniculata* treatment is safe and efficacious for acute respiratory tract infections like common cold and sinusitis. Hence, *A. paniculata*, mainly andrographolide, could be considered as an excellent candidate for antimicrobial drug development. Considering the importance, medicinal values, and significant role as antimicrobial agents, this study critically evaluated the antimicrobial therapeutic potency of *A. paniculata* and its metabolites, focusing on the mechanism of action in inhibiting invasive microbes and biofilm formation. A critical evaluation of the secondary metabolites with the aim of identifying pure compounds that possess antimicrobial functions has further added significant values to this study. Notwithstanding that *A. paniculata* is a promising source of antimicrobial agents and safe treatment for IDs, further empirical research is warranted.

## 1. Introduction

Infectious disease (ID) is a serious global health problem that leads to a high mortality rate worldwide every year [1]. The world has recently witnessed a most formidable threat in recent human history, COVID-19, in the modern era of highest advancement of medical sciences. Infectious agents (i.e., invading microbes or pathogens) evolved a variety of strategies, such as modulating their cell surfaces, releasing proteins to inhibit or degrade host immune factors, or even mimicking host molecules to evade the host immunity and ensuring their own survival within a host [2,3,4]. Pathogens, particularly bacteria, are multifaceted in their methods used to escape host immune detection. Due to having the mastery of these camouflaging and precise weaponry techniques by pathogens, developing new vaccines and innovative treatments become challenging, even in some cases almost impossible, for example, treating antibiotic-resistant strains. As a result, the death toll is increasing rapidly. For example, multidrug-resistant tuberculosis (MDR-TB) has recorded around 206,000 new cases in 2019, a 10% increase from 187,000 in 2018, and a total of 1.4 million people died from tuberculosis in 2019 [5]. Furthermore, antibiotics usage causes some common side effects, including hypersensitivity and depletion of beneficial gut microorganism [6,7].

Acute upper respiratory tract infections (URTIs) is another significant cause of antibiotic resistance since physicians needlessly write antibiotic prescriptions [8,9,10,11,12]. Even though the vast majority of URTIs are mild, self-diagnosed and self-treated, they are the most common reason for absenteeism from school or work [13]. URTIs can be mainly caused by viruses, such as a rhinovirus, influenza virus, adenovirus, enterovirus, and respiratory syncytial virus. Bacteria like *S. pyogenes*, a Group A *Streptococcus,* may cause roughly 15% of sudden onset pharyngitis presentations [8]. Viral pharyngitis is mainly treated based on the symptoms that appeared, whereas bacterial pharyngitis can be treated with antibiotics. However, current evidence does not support the usefulness of antibiotics treatment in non-specific URTIs [13,14]. Therefore, research is urgently required to find alternatives to conventional medications for eradicating IDs. Natural products based therapy could be an excellent source of antimicrobial agents that would offer symptomatic relief since they have the high potentiality to inhibit the growth of microbes in the host-defence mechanism [4], as well as they offer promising outcomes in the scientific investigations [15,16,17,18,19,20]. Additionally, it would reduce unnecessary antibiotic prescription; therefore, the chances of antibiotic-resistance would be reduced.

Nowadays, the philosophy of drug discovery has transformed into “*one drug, multitarget*” from “*one drug, one target*” [16,21,22,23,24,25,26,27,28]. Plant-derived secondary metabolites hold the potential of multi-targeting properties as they need to undergo evolving defence mechanisms of the plant against predators like bacteria, fungi, virus, even insects and herbivores [15,16,21,22,29,30]. A majority of the world population relies on medicinal plants for first-line treatment due to the severe side effects of synthetic drugs [31]. Moreover, plants’ ability to cure diseases and the necessity of their study in sacred texts motivated people to use natural remedies and researchers to study their pharmacology [32,33].

Plant-based secondary metabolites commonly isolated are phenols, tannins, flavonoids, lignans, terpenes, and a wide range of alkaloids [21]. Since natural products are better models with ideal pharmacokinetics/ pharmacodynamics properties [16], often feature biologically relevant molecular scaffolds and pharmacophore patterns that have evolved as preferred ligand-protein binding motif [22], they gained tremendous importance for the development of polypharmacological drugs for IDs, cancers, and neurological disorders [22,34]. Furthermore, about 80% of drugs are either natural products or analogues mimicking them, and steadily increasing approval rate (after the 1990s, the average annual approval rate is 10.3) of natural product-derived drugs from the US Food and Drug Administration (FDA) have encouraged researchers and pharmaceutical industries to search the effective multitarget drugs for various ailments [30,34]. Currently, a number of natural products, including morphine, quinine, reserpine, cocaine, and ephedrine, are now available in pure form as drug substances [15]. Besides, many pure compounds are identified by pharmaceutical scientists worldwide because of having advanced technology that eases and fasten the characterization and structural elucidation of isolated metabolites. Capitalizing on these findings are crucial for medical advancement to overcome unavoidable circumstances happed by synthetic drugs.

One attractive medicinal plant and its metabolites that have gained considerable and progressive interest for decades are *Andrographis paniculata* (Burm. f.) Wall. ex Nees. This annual plant belongs to the *Acanthaceae* family and is commonly known as ”*King of the bitters*” or ”*Kalmegh*”. It is native to India and Sri Lanka and widely found in Southern and Southeastern Asia, including Bangladesh, China, Hong Kong, Indonesia, Malaysia, Myanmar, Philippines, and Thailand [35,36,37,38,39]. Usually, the aerial parts, roots or leaves of *A. paniculata* are used separately. These plant parts are used traditionally as powder, infusion, or decoction form either alone or in combination with other medicinal plants for the treatment of leprosy, gonorrhoea, respiratory tract infections, scabies, boils, skin eruptions, chronic and seasonal fevers, griping, irregular bowel habits, loss of appetite, alopecia, general debility, diabetes, jaundice, dyspepsia, hemopathy, cough, oedema, liver complaints, dysentery, malaria, enteritis, helminthiasis, herpes, peptic ulcer, skin infections (topical use), and snake-bites (topical use) [35,36,39,40].

Modern science is focusing on validating the traditional claims of this plant through systemic investigations. Different extracts (i.e., acetone, chloroform, ethanol, hexane, methanolic or aqueous extract) and isolated pure metabolites from *A. paniculata* have been investigated for pharmacological properties, for example, antibacterial, antiviral, antifungal, antiparasitic, choleretic, hypocholesterolemia, anti-inflammatory, anti-hyperglycemic, hepatoprotective, anticancer, immunomodulatory, cardiovascular, antihyperlipidemic, emollient, anti-snake venom, anti-platelet aggregation, anti-fertility, carminative, and antipyretic properties at in vitro and in vivo conditions [41,42,43,44,45,46,47,48,49,50,51,52,53].

Clinical trials were conducted to validate the in vitro and preclinical antimicrobial pharmacology of *A. paniculata*. Several clinical trials have justified the anti-infectious activity of *A. paniculata* against URTIs, influenza, and HIV as well as its effectiveness in treating osteoarthritis and multiple sclerosis [54,55,56,57,58,59]. The clinical studies conducted using the standardized *A. paniculata* extracts alone or in combination with other medicinal plants (i.e., Kan Jang, KalmCold™). The extracts reported having approximately 4–6% of andrographolide [56,60], responsible for the beneficial effects of the plant extracts. In recent years, there are few more clinical studies conducted [12,61,62,63,64,65,66,67,68,69,70,71,72,73]. However, to date, Andrographolide or *A. paniculata* extracts have not achieved the human clinical trial stage. This might be because of low bioavailability that is attributed to the fast biotransformation and removal from the body [74] or due to a lack of a well-defined mechanism of action because it is considered as a highly promiscuous compound and engaged in covalent interactions with numerous previously unknown cellular targets in cell type-specific manner [75]. There are two important and typical challenges: (i) poor pharmacokinetics and (ii) limited bioavailability are involved with many natural products, for example, β-sitosterol, quercetin, genistein, rutin etc., prevent their in vitro to preclinical or clinical translation [76]. Nevertheless, there is a numerous investigation reported to have potential efficacy of *A. paniculata* for IDs as well as other ailments, including clinical efficacy. So, would *A. paniculata* extracts and their metabolites be a choice of better therapeutics? Would it be undergone for clinical development? Hence, there is a need to explore further the feasibility of *A. paniculata* with potential use as anti-infective agents for a wide range of invasive microbes. A better understanding of the mode of actions of the active ingredients of *A. paniculata* would enhance the clinical development of this drug. Additionally, there were several recent in vitro, in vivo, and a few clinical trials reported its potential therapeutic efficacy for antimicrobial pharmacology. To our best knowledge, critical evaluation of *A. paniculata*’s purported benefits is not substantial yet; hence, it would be important to review them before conducting further research in this area.

Therefore, the objectives of this study were: (i) to establish a comprehensive list of antimicrobial metabolites of *A. paniculata*, (ii) to discuss the antimicrobial therapeutic potency of *A. paniculata* and its metabolites, focusing on the mechanisms of actions, (iii) to evaluate the salient achievements of controlled clinical trials systematically to determine its efficacy and safety for the treatment of infections or infection causing agent.

## 2. *Andrographis paniculata*—“The King of Bitter”

### 2.1. Botanical Data of A. paniculata

*A. paniculata* is an economically important herb of the *Andrographis* genus. It is an erect and branched and annual flowering herb. This herb grows well in hedgerows throughout the plane lands, hill slopes, waste ground, farms, moist habitat, seashores, and roadsides [38,39]. It can also be grown in the garden as well. For their excellent development, moist and shady places, forests, and wastelands are preferable. It is a salt-sensitive plant [77]; therefore, its growth is limited under stress conditions, particularly salinity stress that drastically affects plant growth and crop productivity [78,79].

**Figure 1:**
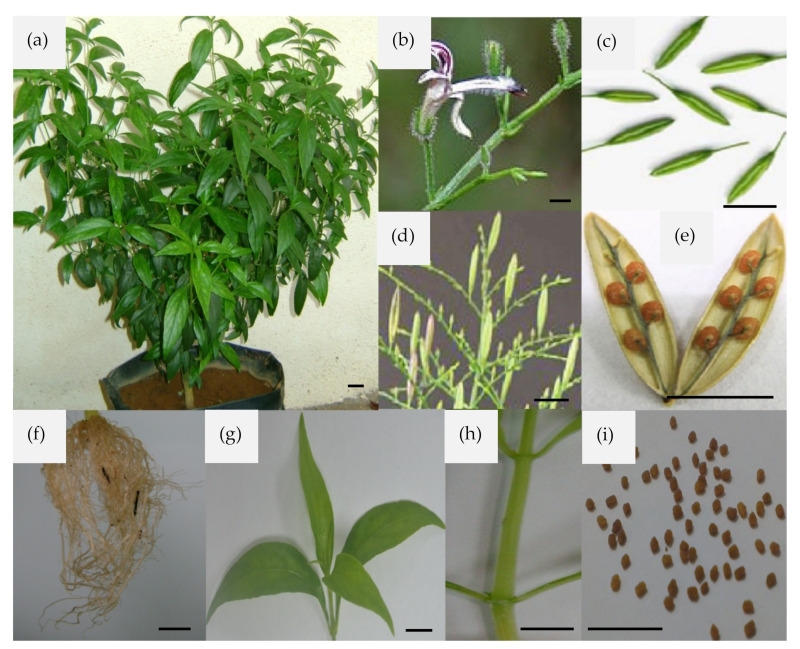
*Andrographis paniculata* and its different parts. (**a**) Aerial part, (**b**) flower, (**c**) pod stage with panicles: mature capsule, (**d**) fruit, (**e**) opened capsule, (**f**) roots, (**g**) leaves: opposite arrangement, (**h**) stem, and (**i**) seed. Bar = 1 cm. This figure is reproduced from a thesis of the first author of this article [77].

This plant, under cultivation, can reach up to a height of 30 to 110 cm. Its stem is dark green, 30–110 cm in length, 2 to 6 mm in diameter, quadrangular with longitudinal furrows and wings at angles of the young parts, slightly enlarged at the nodes. The leaves are dark green, glabrous, 2–12 cm long and 1–3 cm broad, opposite, decussate, lanceolate, entire margin, and venation pinnate; the petiole is very short. The flowers are small, consist of five linear particle calyx, narrow tube, and about 6 mm long white corolla with rose-purple spots on the petals. December to April is the flowering and fruiting period. The fruits are small 2-celled odourless erected capsules, 1–2 cm long, 2–5 mm wide, linear-oblong, acute at both ends and compressed. The leaf taste is intensely bitter, and seeds are numerous, sub-quadrate and yellowish-brown (Figure 1) [77]. The detailed botanical description has been reviewed somewhere else [39]. Different extracts and their secondary metabolites, particularly andrographolide, are one of the extensively studied natural products. The therapeutically active extracts are prepared, or metabolites isolated from aerial parts, leaves, roots, whole plants (Figure 1) or callus [36,37,80].

### 2.2. Recent Progress in Publication of A. paniculata

**Figure 2:**
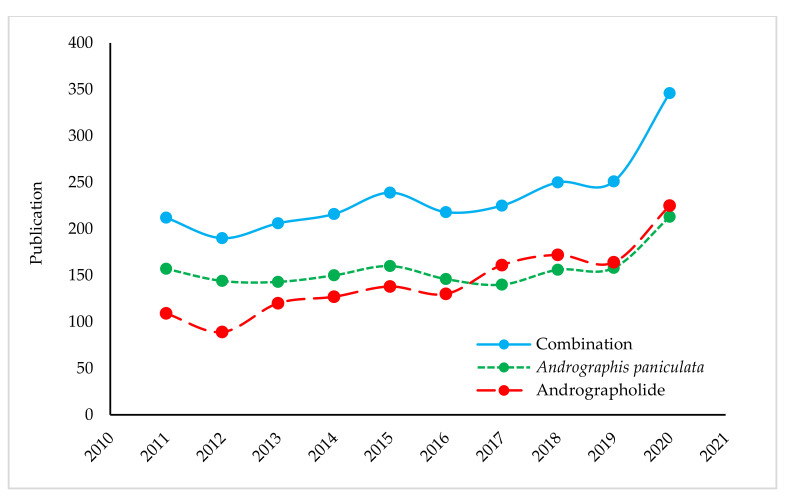
Annual publication statistics on topics covering the *A. paniculata* and andrographolide from 2011 to 2020 (Scopus).

The measurement of scientific interest in a particular topic can be revealed from its trend of publications. Due to having tremendous medicinal importance, the relentless interest in this plant and its versatile molecules have resulted in overwhelming publications over the past ten years. The publication numbers amounted to 3279 (as of 15 February 2021). In other words, we can say, daily, almost one publication (Figure 2), of which about 14% publications were about the antimicrobial study of either *A. paniculata* extracts or its metabolites, especially andrographolide. This bibliometric data was extracted from the Scopus database using the query of the term “*Andrographis paniculata*” OR “andrographolide” in titles, abstracts, and keywords. This number might be increased if data can be combined from different databases like PubMed, Web of Science and so on.

## 3. Invasive Microbes Used in the Antimicrobial Study of *A. paniculata*

There are about 1400 known species of human pathogens. Although this seems like a large number, they are less than 1% of the total number of microbial species on the planet earth [81]. Even though this is less than 1% of total microorganisms, a harmless microbe can sometimes be harmful under a specific condition like an immunocompromised patient. Exploring proper medications for these spontaneous behavioural changing microbes is a continuous effort of the scientific community. *A. paniculata* extracts and their bioactive molecules were investigated against a wide variety of pathogens, including several antibiotic-resistant species, for example, *Staphylococcus aureus*, *Pseudomonas aeruginosa*, *Shigella* spp., *Salmonella* spp., *Candida* spp., *Streptococcus pneumoniae*. We have identified a total of 59 invasive microbes that have been used to investigate the antimicrobial efficacy of *A. paniculata* extracts and/or their isolated pure compounds. The categorized microbes included 33 bacterial, four viral, 12 fungal and ten parasite species. The details antimicrobial effectiveness of different extracts has been discussed in the later section. The list of tested microbes, their types, mode of transmission, a disease caused, and infecting organisms are presented in the Supplementary Table S1.

## 4. Antimicrobial Secondary Metabolites of *A. paniculata*

*A. Paniculata* contains therapeutically active secondary metabolites that include lactones, diterpenes, flavonoids, quinic acid, xanthones, noriridoids, and other compounds. In our previous study [39], we reported more than 55 *ent*-labdane diterpenoids, 30 flavonoids, eight quinic acid derivatives, four xanthones, and five rare noriridoids in *A. paniculata*; however, in this study, our extensive review revealed at least 142 secondary metabolites that already isolated from *A. paniculata* using different plant parts and fractionations of organic solvents (i.e., acetone, butanol, chloroform, ethanol, methanol, and hexane) or water and chromatographic analysis like thin layer chromatography (TLC), high-performance thin-layer chromatography (HPTLC), liquid chromatography, micellar electrokinetic capillary chromatography (MECC), high-speed counter-current chromatography (HSCCC), high-performance liquid chromatography (HPLC), ultra-performance liquid chromatography (UPLC), Silica Gel Chromatography (SGC), and flow injection spectrophotometry (FIS) [65,80,82,83,84,85,86,87,88,89,90,91,92,93,94,95,96,97,98,99,100,101,102,103,104,105,106,107,108,109,110,111,112,113,114,115,116,117,118,119,120,121,122,123,124,125,126,127,128,129,130,131,132,133,134,135,136,137,138,139,140,141,142,143,144,145,146,147,148,149,150,151,152,153,154,155,156,157,158,159,160,161,162,163,164,165,166,167,168]. Among the chromatographic methods, HPLC and TLC are more commonly used. This might be due to easy accessibility and accuracy. The UPLC is good for its selectivity, linearity, precision, accuracy, stability, robustness, the limit of detection and quantification. This method showed good linearity, accuracy and satisfactory precision with a run time of less than 3 min [165].

The identified secondary metabolites included 78 *ent*-labdane diterpenoids, 41 flavonoids, eight quinic acid derivatives, four xanthones, five rare noriridoids, three steroids and three other compounds (Supplementary Table S2). A recent study also reported a similar number of unique secondary metabolites [169]. Among these compounds, about 80 compounds showed different types of pharmacological activities, including antibacterial [109,139,170,171,172,173,174,175], anti-biofilm [88,176], antiviral (i.e., chikungunya virus [91], Anti-HIV [177,178,179], anti-influenza [99,180], *Herpes simplex* virus 1 [83,99,181], dengue virus serotype 1 [92,93,94,182], anti-Epstein Barr virus [97], pestivirus and flavivirus [90], human papillomavirus type 16 [96]), anti-fungal [109,183], antiparasitic (i.e., antimalarial [95,184,185], anti-Leishmaniasis [98]), antiproliferative [85,86,87,120,122,124,127,128,177,186], cytotoxicity [84,85,86,101,102,127,186], anti-inflammatory [84,86,136,187], antiplatelet aggregation [102], phagocytosis and anti-complement [84,86,102,104,109,113,114,117,128,133,135,138,142,177]. Some of these metabolites did not exhibit pharmacological properties or showed very week effectiveness. The leads secondary metabolites isolated mainly from aerial parts (majority cases), leaves, whole plant, and some from roots (Supplementary Table S2). The part used, geography, season, and time of harvesting of plant materials significantly influences the quantity and quality of phytoconstituents [39,188].

**Figure 3:**
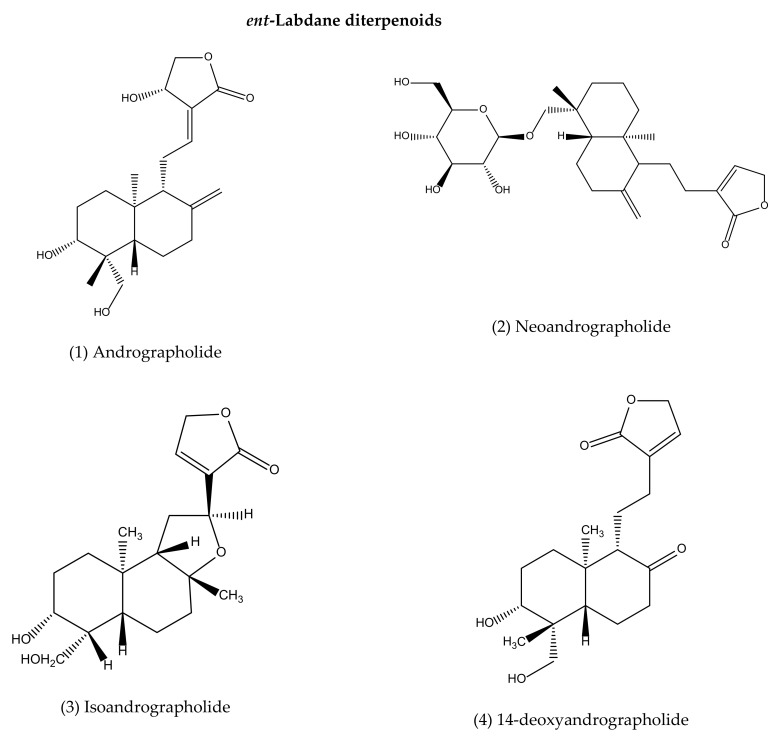

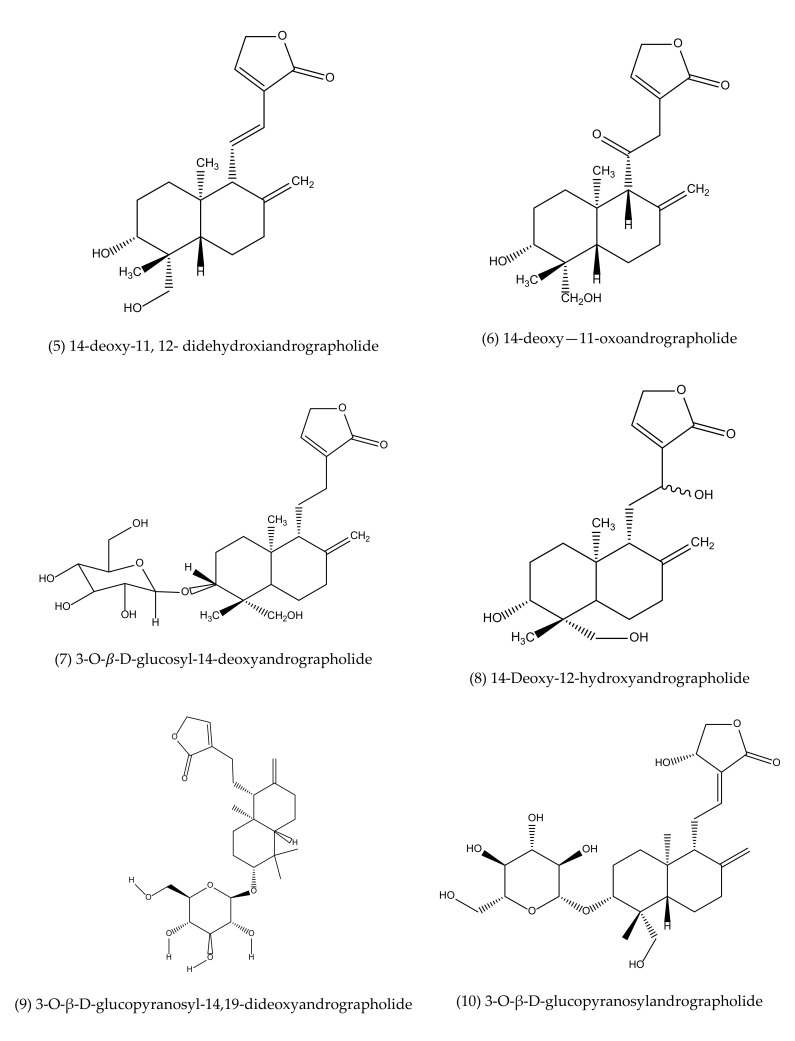

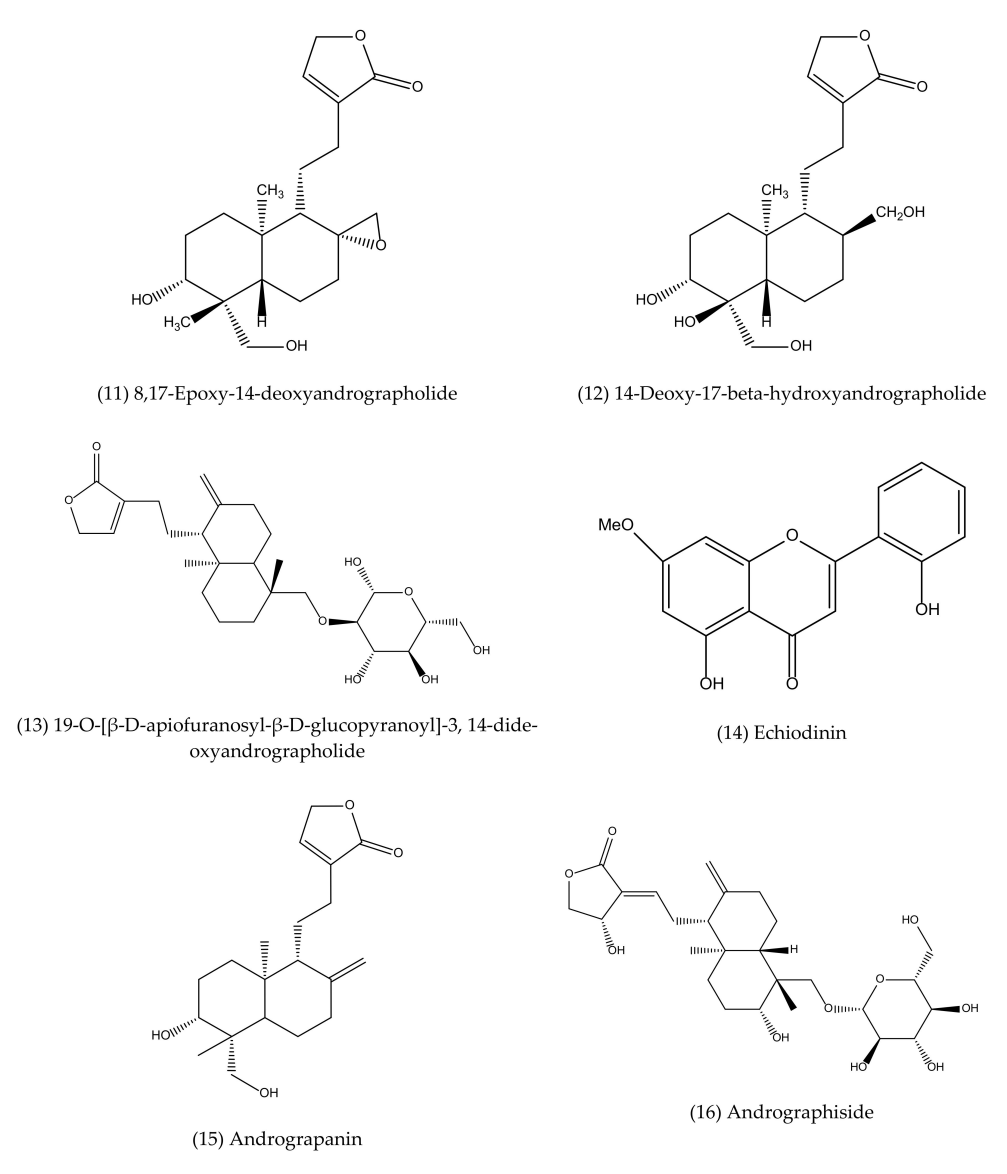

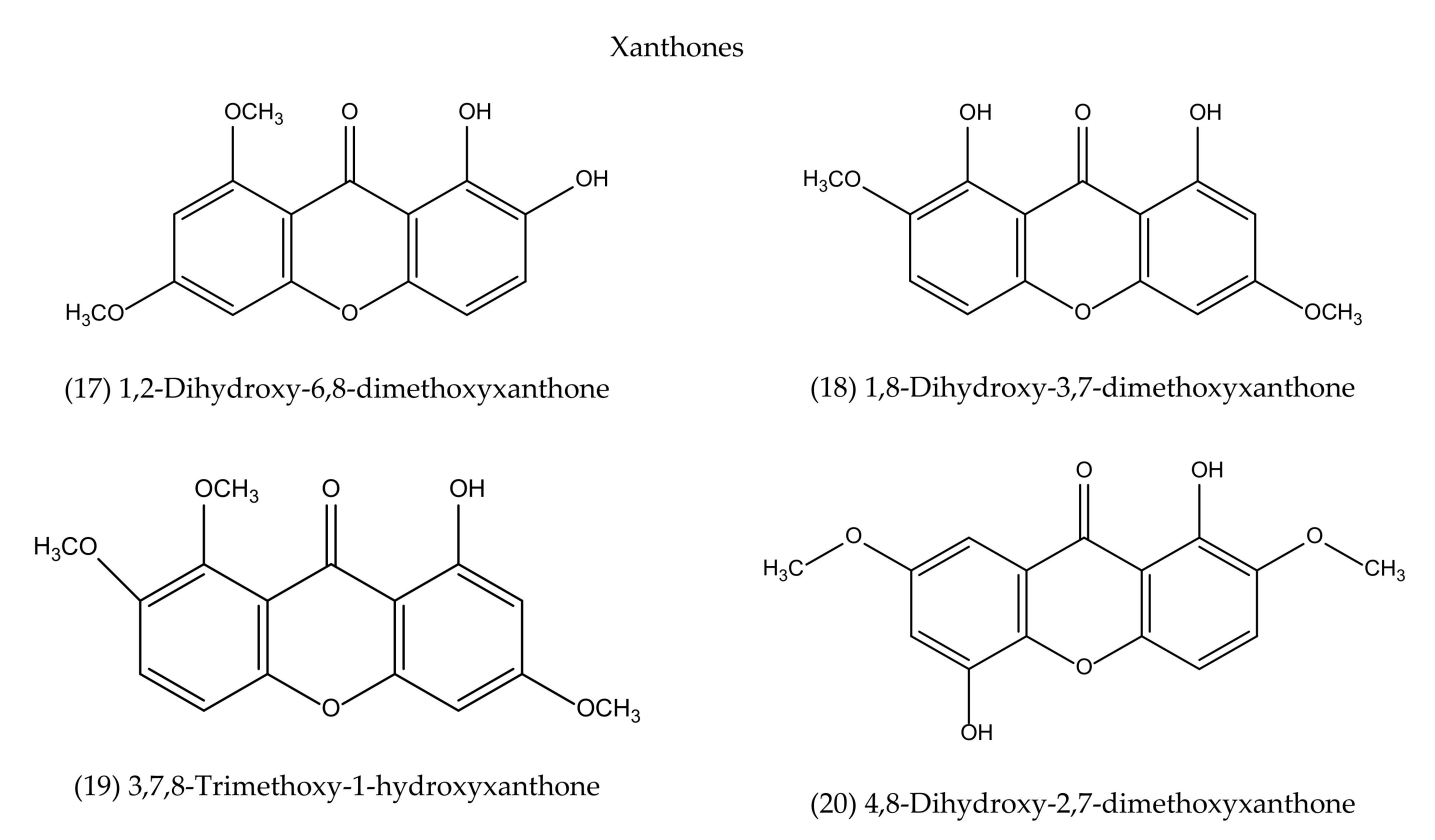
Antimicrobial agents (pure compounds) of *A. paniculata* [39,176,192,193].

**Figure 4:**
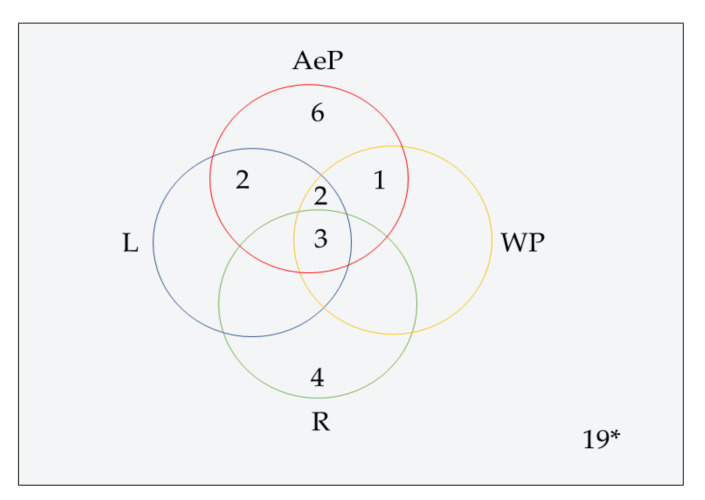
Venn diagram shows antimicrobial pure metabolites source of part of *A. paniculata*. AeP: Aerial part, L: Leaves, R: Root, WP: Whole plant. * One metabolite (14) isolated from callus, which is not shown in the diagram.

To our best knowledge, a total of 35 isolated compounds have tested for antimicrobial activities, of which 20 secondary metabolites (Figure 3) showed antimicrobial effects (Table 1). Antimicrobial metabolites were extracted from the whole plant, aerial part, leaves and roots (Figure 4). Most of the lead compounds have high potential, particularly compounds (1–7, 14–20) against microbes. However, few compounds (8–13) showed very weak potentiality or were inactive against different strains of bacteria, for example, *Bacillus subtilis*, *Staphylococcus aureus*, *Escherichia coli*, *Micrococcus luteus*, *Sarcina lutea* and fungi, such as *Candida albicans*, *Candida sake*, and *Aspergillus niger* [109].

Andrographolide (1), neoandrographolide (2) and isoandrographolide (3) are the most abundant lead bioactive compounds that can be isolated from any part of *A. paniculata* for example, aerial apart, leaves, whole plant, and even roots. However, these present in high amounts in leaves [82]. The yield reached the maximum level while the plant materials collected between 110–130 days of cultivation [189]. Compounds 4 and 5 are the next most abundant, followed by 6, 7, 14, 15. The least abundant are compounds 17–20, which are only available in roots [190,191].

**Table 1:** Isolated secondary metabolites of *A. paniculata* and their antimicrobial activity.

No.	Name	Sources	Extraction Solvent	Analytical Technique	Antimicrobial Potentiality
***ent-*Labdane diterpenoids**					
**1**	Andrographolide	AeP, L, R, WP	AW, E, H, M	HPLC, HPLC-MS, MECC, FIS, UPLC, HPLC-DAD	Antibacterial [139,170,171,172,173], anti-biofilm [88,139], anti-CHIKV [91], Anti-HIV [177,178,179], anti-influenza [99,180], anti-HSV-1 [83,99,181], anti-DVS-1 [92,93,94,182], anti-EBV [97], anti-HPV-16 [96], anti-HBV [186], anti-HCV [194], antimalarial [95,184,185], anti-Leishmaniasis [98], pestivirus and flavivirus [90]
**2**	Neoandrographolide	AeP, L, R, WP	AW, E, M	TLC	Anti-HSV-1 [99], antimalarial [184]
**3**	Isoandrographolide	AeP, L, R, WP	AW, E, M	HPLC, TLC	Antibacterial [109,174,175], anti-fungal [183]
**4**	14-deoxyandrographolide	AeP, L, WP	AW, E, H, M	HPLC, TLC	Antibacterial [174,175], anti-fungal [183], antimalarial [109,184]
**5**	14-deoxy-11, 12-didehydroxiandrographolide	AeP, L, WP	E, H, M, DCM	HPLC, TLC	Antibacterial [109], anti-biofilm [88], Antifungal [109,183], anti-HIV [177], Anti-HSV [99]
**6**	14-deoxy—11-oxo- andrographolide	AeP, L	AW, M	SGC	Anti-Leishmaniasis [89,167]
**7**	3-O-β-D-glucosyl-14-deoxy- andrographolide	AeP, WP	E, M	HPLC, TLC	Antibacterial [174,175], anti-fungal [183]
**8**	14-Deoxy-12-hydroxy- andrographolide	AeP	AW, M	HPLC, TLC	Antibacterial [109], anti-fungal [109]
**9**	3-O-β-D-glucopyranosyl- 14,19-dideoxyandrographolide	AeP	AW, M	HPLC, TLC	Antibacterial [109], anti-fungal [109]
**10**	3-O-β-D-glucopyranosyl- andrographolide	AeP	AW, M	HPLC, TLC	Antibacterial [109], anti-fungal [109]
**11**	8,17-Epoxy-14-deoxy-andrographolide	AeP	AW, M	HPLC, TLC	Antibacterial [109], anti-fungal [109]
**12**	14-Deoxy-17-β-hydroxy- andrographolide	AeP	AW, M	HPLC, TLC	Antibacterial [109], anti-fungal [109]
**13**	19-O-[β-D-apiofuranosyl-β-D-glucopyranoyl]-3,14-dideoxy- andrographolide	AeP	AW, M	HPLC, TLC	Antibacterial [109], anti-fungal [109]
**14**	Echiodinin	Callus	AW, M	TLC	Antibacterial [80]
**15**	Andrograpanin	AeP, L	E, H	HPLC, TLC, SGC	Antibacterial [109], anti-fungal [109], Antibiofilm [168,176]
**16**	Andrographiside	WP	n-butanol	TLC	Antimalarial [184]
**Xanthones**					
**17**	1,2-Dihydroxy-6,8-dimethoxyxanthone	R	CF, M, PE, W	TLC	Antimalarial [190,191]
**18**	1,8-Dihydroxy-3,7-dimethoxyxanthone	R	CF, M, PE, W	TLC	Antimalarial [190,191]
**19**	3,7,8-Trimethoxy-1-hydroxyxanthone	R	CF, M, PE, W	TLC	Antimalarial [190,191]
**20**	4,8-Dihydroxy-2,7-dimethoxyxanthone	R	CF, M, PE, W	TLC	Antimalarial [190,191]

AeP: Aerial part, AW: Acetone-water, CF: Chloroform, CHIKV: Chikungunya Virus, DCM: Dichloromethane, E: Ethanol, H: Hexane, L: Leaves, M: Methanol. PE: Petroleum ether, R: Root, W: Water, WP: Whole plant, HSV: Herpes Simplex Virus, DVS: Dengue Virus Serotype, EBV: Epstein Barr virus, HPV: Human Papilloma Virus, MECC: micellar electrokinetic capillary chromatography, FIS: flow injection spectrophotometry, TLC: Thin Layer Chromatography, HPLC: High-performance Liquid Chromatography, HPLC-DAD: HPLC with a diode-array detector, HPLC-MS: HPLC-Mass Spectrometry, UPLC: Ultra-performance Liquid Chromatography, SCG: Silica Gel Chromatography.

## 5. Antimicrobial Pharmacology

### 5.1. Antibacterial Effects

For centuries, the traditional use of *A. paniculata* in treating several IDs caused by bacteria encouraged researchers to study its anti-bacterial properties and how it fights against invasive microbes. Our review found about 33 different types of bacteria that were inhibited by different types of extracts (Table 2 and Supplementary Table S1).

#### 5.1.1. *A. paniculata* Extracts as Antibacterial Agents

Researchers used different types of extracts of *A. paniculata* to explore their potentiality against numerous invasive microbes, including antibiotic-resistant strains, such as methicillin-resistant *S. aureus* (MRSA), vancomycin-resistant *E. faecalis* (VRE), carbapenem-resistant *Actinobacillus baumannii*, β-Lactamase-negative, ampicillin-resistant (BLNAR) *Haemophilus influenzae*, *P. aeruginosa*. A summary of the antibacterial activity of the different types of *A. paniculata* extracts is shown in Table 2.

The aqueous extract of *A. paniculata* showed significant antibacterial activity, which was further linked to the presence of andrographolides and arabinogalactan proteins [195]. The role of andrographolide and neoandrographolide in treating bacillary dysentery caused by *Shigella* sp. was reported in several studies as well [196,197]. In an experiment, *A. paniculata* was used to treat 1,611 cases of bacterial dysentery and 955 cases of diarrhoea [198]. The efficacy of *A. paniculata* extracts for dysentery was proved from laboratory stool test with 82.5% and 91.3% [198,199]. However, the crude water extract of *A. paniculata* leaves exhibited no effect on *E. coli* and *K. pneumoniae*. A significant activity against the Gram-positive *S. aureus,* MRSA, and Gram-negative *P. aeruginosa* was reported by crude water extracts of leaves sample [200].

In contrast, methanol extract of leaves showed significant activity against *E. coli* along with *P. aeruginosa, K. pneumonia, S. aureus, B. subtilis* and *Streptococcus epidemidis* [201]. Furthermore, no antimicrobial effect of the aqueous extracts of the whole plant and isolated andrographolide on tested common pathogenic bacteria [170]. However, ethanol extracts were found effective against *Legionella pneumophila* and *Bordetella pertussis* only [170]. These findings indicated that the extraction process and solvent have a significant role in the efficacy of *A. paniculata* as the number and yield of pure metabolites greatly differ depending on the types of fractions.

Significant antibacterial activity of subsequent hexane, chloroform, n-butanol, and aqueous fractions of 50% ethyl alcohol-treated extracts of *A. paniculata* exhibited against *E. coli* [202]. Similarly, the potent inhibitory effect of ethanol extract of aerial parts on the growth of both gram-positive and gram-negative bacteria, namely, *Salmonella typhi*, *V. cholera*, *V. alginolyteus*, *S. aureus*, *Shigella boydii*, *Shigella sonnei*, *E. coli*, *B. licheniformis*, and *Salmonella typhimurium*. Another study [173] reported that the co-presence of andrographolide and arabinogalactan proteins in the ethanol extract was further acknowledged for its enhanced antibacterial activity compared to andrographolide and arabinogalactan proteins alone. This outcome has been derived maybe because of their synergistic effect.

**Table 2:** Antibacterial activity of the different types of *A. paniculata* extracts.

**Plant Part**	Extraction Methods	Assay	Number of Test MOs	Most Inhibited Mos	MEIC	ZOI (mm or %)	Remarks	Reference
AeP	Chloroform	AWDM	9	*Enterobacter faecalis*	250 μg/mL	35	Seven out of 9 pathogens were inhibited that were comparable with antibiotic, amikacin	[203]
L	Water	DDM	5	*P. aeruginosa* *S. aureus* MRSA	2 µg/disc 1000 µg/disc 250 µg/disc	8 ± 0.1 6 ± 0.1 8 ± 0.1	No activity against *K. pneumoniae* and *E. coli.*	[200]
L	70% Methanol	Two-fold broth MDM	10	*Edwardsiella tarda* *E. coli* *Flavobacterium* sp. *P. aeruginosa* *Vibrio cholerae*	31.5 mg/L	-	All the test MOs were inhibited	[204]
WP	DCM	DDM	12	*E. faecalis* *S. aureus* *S. saprophyticus*	1000 µg/disc	21.33 ± 1.53 20.00 ± 1.50 19.33 ± 1.15	Gram-negative bacteria were more resistant.	[205]
WP	Methanol			*E. faecalis* *S. aureus* *S. saprophyticus*	1000 µg/disc	24.00 ± 0.00 22.00 ± 0.00 22.00 ± 1.53	No activity observed against *S. saprophyticus* at 250 µg/disc	
WP	Aqueous			*M. luteus* *S. pyogenes* *E. faecalis*	1000 µg/disc	23.17 ± 0.76 22.67 ± 0.58 22.00 ± 1.00	No activity was observed against *M. luteus, S. pyogenes, E. faecalis* and *K. pneumoniae* at 250 µg/disc	
R	Hexane	Broth MDM	4	*B. pumilus* *B. subtilis* *E. coli* *Proteus vulgaris*	100 mg/mL 100 mg/mL 200 mg/mL 200 mg/mL	12 12 13 12	Hexane and methanolic extracts were more efficient against all tested MOs	[206]
R	Methanol	Broth MDM	4	*E. coli* *B. subtilis* *Proteus vulgaris*	100 mg/mL 200 mg/mL 200 mg/mL	12 12 13		
WP	Methanol	CPADM	5	*S. aureus*	1000 μg/mL	19.67 ± 0.76	Gram-negative bacteria were more resistant to methanol extracts	[174]
AeP	Ethanol	AWDM	11	*S. typhi* *V. cholerae*	200 μg/mL	14 13	The ethanol extract was efficient	[173]
L	Methanol	AWDM	6	*S. aureus*	50 mg/mL	24 ± 0.2	Inhibit both Gram-positive and negative bacteria, but gram-negative bacteria are less susceptible	[201]
WP	DCM	DDM	10	*S. aureus*	1000 μg/disc	20 ± 1.50	Aqueous extracts were more effective compared to the DCM and methanol extracts	[207]
	Methanol			*S. aureus* *S.saprophyticus*	1000 μg/disc	22 ± 0.00 22 ± 1.53		
	Aqueous			*M. luteus*	1000 μg/disc	23.17 ± 0.76		
WP	Methanol	CPADM	5	*S. aureus* *M. luteus*	1000 μg/mL	19.67 ± 0.76 18.50 ± 0.58	Effective against all test MOs	[175]
L	Chloroform	AWDM	6	*B. subtilis*		22 ± 0.071	Chloroform extract of leaves was more efficient to inhibit all tested MOs than other extracts	[208]
	Aqueous		6	*K. pneumoniae* *S. aureus* *B. subtilis*		12 ± 0.344 12 ± 0.447 12 ± 0.084		
	Acetone		6	*S. aureus*		13 ± 0.416		
	Ethyl acetate		6	*B. subtilis*		15 ± 0.152		
	Petroleum ether			*-*	-	-	No inhibitory activity	
R	Chloroform	AWDM	6	*B. subtilis*		18 ± 0.055	Chloroform extract of roots was more efficient to inhibit all tested MOs than other extracts	[208]
	Aqueous			*K. pneumoniae*		14 ± 0.297		
	Acetone			*S. aureus*		15 ± 0.055		
	Ethyl acetate			*B. subtilis*		10 ± 0.626		
	DMSO			*S. aureus*		14 ± 0.187		
	Petroleum ether			*-*	-	-	No inhibitory activity	
S	Ethyl acetate	AWDM	6	*S. aureus* *B. subtilis*		8 ± 0.303 8 ± 0.327	Chloroform extract of stems was more efficient to inhibit all tested MOs than other extracts	[208]
	DMSO			*S. aureus*		16 ± 0.332		
	Acetone			*S. aureus*		16 ± 0.374		
	Chloroform			*B. subtilis*		24 ± 0.219		
	Aqueous			*B. subtilis*		13 ± 0.373		
	Petroleum ether			*-*	-	-	No inhibitory activity	

AeP: Aerial part, L: Leaves, R: Root, S: Stem/bark, WP: Whole plant, AWDM: Agar Well Dilution Method, CPADM: Cup-plate Agar Diffusion Method, DCM: Dichloromethane, DDM: Disc Diffusion Method, MEIC: Most Effective Inhibitory Concentration, DMSO: Dimethyl Sulfoxide, FA: Fluorogenic Assay, MDM: Microdilution Method, MOs: Microorganisms, NA: No activity, ZOI: Zone of Inhibition.

#### 5.1.2. Isolated Compound as Antibacterial Agent: Mechanisms of Action

A substantial number of evaluations proved the efficacy of different extracts of *A. paniculata* against many severe pathogenic microbes. Besides this, 13 pure secondary metabolites of *A. paniculata* were also reported to have significant antibacterial effects (Table 1). These are compounds 1, 3–5, and 7–16. These compounds have been used to evaluate antibacterial potency against a wide range of bacteria. Overall, Gram-positive bacteria were more susceptible to compound 1 than Gram-negative bacteria due to the presence of the outer membrane and the polarity nature of the compound [119,139]. Depending on the bacterial species, the mode of actions of compound 1 differs by a large extent. *S. aureus* was largely susceptible (MIC is 0.1 mg/mL) to compound 1 among the tested microbes [139]. Healthcare-associated infections are prevalently (10.7%) caused by *S. aureus*, a major bacterial human pathogen that can form biofilm [209]. It causes a wide variety of clinical manifestations, including pneumonia, mastitis, osteomyelitis, endocarditis, skin infections, abscesses, food poisoning, toxic shock syndrome, and sepsis, and treatment remains challenging due to the emergence of multi-drug resistant strains such as MRSA [210].

**Figure 5:**
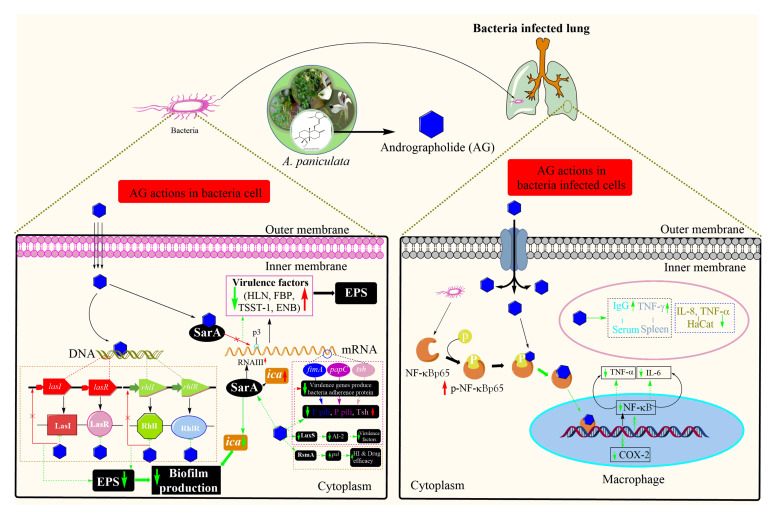
Schematic representation of a plausible mechanism of actions of andrographolide in bacteria, mainly *Staphylococcus aureus*. AG: andrographolide (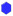), the Black arrow (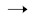): regular process/pathway, Black dotted rectangle area: AG Induction of Salmonella-specific cell-mediated immune response in *Salmonella typhimurum*, Blue dotted rectangle area: AG inhibits cellular inflammatory factor in *Propionibacterium acnes*, COX-2: Cyclooxygenase-2, ENB: Enterotoxin B, FBP: Fibronectin-binding protein, Green dotted arrow (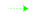): expected regulation process, Green dotted arrow with a flat bottom (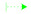): attachment of AG to the target site, Green down arrow (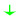): downregulation and expected outcomes, Green up arrow (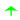): upregulation and expected outcomes, HaCaT: Human Epidermal Keratinocyte line, HLN: Hemolysin, IFN-γ: Interferon-gamma, IgG: Immunoglobulin G, IL-6: Interleukin 6, IL-8: Interleukin 8, NF-κB: Nuclear factor-kappa-light-chain-enhancer of activated B cell, Orange dotted rectangle area: AG analogue influences the quorum sensing system and inhibits exopolysaccharides generation in *Pseudomonas aeruginosa*. *psl* production is also significantly inhibited in this biofilm-forming bacteria, (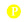): Phosphorylation, Purple dotted rectangle area: AG influences quorum sensing system and reduces the expression of F1 pili, P pili and Tsh by downregulating *fimA*, *papC* and *tsh* in *Escherichia coli*. All these virulence genes help bacteria to the adherence cell surface. The red arrow (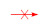): Inhibition/downregulation of the process, Red up arrow (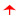): upregulation (at disease or infection stage), TNF-α: Tumor Necrosis Factor- alpha, TSST-1: Toxic Shock Syndrome Toxin-1.

Compound 1 acts on bacteria themselves as well as plays an important role in the regulation of host immunity by regulating macrophage phenotypic polarization and Ag-specific antibody production [211]. When *S. aureus* infected the lungs, it significantly promotes NF-κB p65 phosphorylation and increases TNF-α and IL-6 production (Figure 5). Compound 1 can downregulate them sufficiently but retain the immune cells at the level that can kill bacteria without serious immune damage. In comparison to penicillin, compound 1 showed better management of bacterial infection and persistent host immunity [212]. Since compound 1 works on immune regulation, there are fewer chances of drug resistance. Therefore, compound 1 would further reduce the problems associated with antibiotic resistance, one of the current severe health crises.

In another study, Banerjee et al. [139] reported that compound 1 could strongly inhibit DNA synthesis (approximately 31%) and consequently RNA (about 26% inhibition) and protein (around 36% inhibition) synthesis in *S. aureus*. This result was similar to that of antibiotic ciprofloxacin (25% incorporation). However, cell wall biosynthesis was not hampered [212]. Secondary metabolites showing antimicrobial can work on a specific target site. Compound 1 can affect the quorum sensing system (QSS), a communication system between bacteria; thereby, it is an effective antibacterial target. This system enhances the production of biofilm by bacteria, such as *P. aeruginosa*. The antibacterial drug effects in this system resulting in regulates the production of bacterial efflux pumps and virulence factors [213]. Compound 1 effects on the QSS, especially Las and Rhl systems, resulting in reduced production of compositions of extracellular polymeric substance (EPS), such as carbohydrate, nucleotide, and amino acid polymers, as well as inhibiting virulence factors (Figure 5) [214,215]. In addition, compound 1 could restore the antibiotic sensitivity in *P. aeruginosa* by reducing expression of *mexAB-oprM* efflux pump [216] and inhibit bacterial adhesion, such as *E. coli* and *S. epidermidis,* to the epithelial cells of lungs; therefore, significantly reduced respiratory colonization and level of *fimA*, *papC*, and *tsh* (Figure 5) [217].

#### 5.1.3. Mechanisms of Action Influence on Biofilm Production by Pure Compounds

Among the selected metabolites, compound 1, 5 and 15 exhibited significant anti-biofilm effects against *P. aeruginosa* [88,176] and *S. aureus* [139]. Biofilm is a critical part of bacterial pathogenesis in the host. The multilayer structure of biofilm acts as a potential barrier against the host defense and antibiotics action and serves as a sign of antibiotic resistance as well. Biofilm-embedded bacteria are helped to break free from the drugs and weaken as a result [218]. Biofilm forming infections are getting severe health issues daily and significantly enhancing antibiotic resistance; some are relevant to implant-associated infections [219,220]. Exploring efficient drugs are crucial as conventional antibiotics are not sufficient to inhibit the production of biofilm. *A. paniculata* brought an excellent opportunity to treat biofilm infections as some of its metabolites, for example, compound 5 and 15, possess significant potentiality in inhibiting biofilm formation. Interestingly, *A. paniculata* metabolites showed synergistic effects with standard antibiotics (i.e., gentamicin and azithromycin), which were unable to reduce biofilm growth alone [88,176]. Compound 5 and 15 showed efficacies in a dose-dependent manner. Compound 15 at 0.125 mM concentration prevented about 54% biofilm formation. Inhibition of biofilm production was further augmented up to 60% while the concentration was used at 0.15 mM [176]. Similarly, compound 5 showed a significant reduction of biofilm growth (about 56%) by *P. aeruginosa*, whereas compound 1 exhibited only 40% inhibition of biofilm production [88]. Neither gentamicin nor azithromycin was incapable of inhibiting biofilm production of *P. aeruginosa*. However, the outcome was dramatically changed to about 90% inhibition (*p* < 0.0001) when the bacteria were treated in a combination of compound 15 and gentamicin [119]. In compound 5, this inhibition becomes about 92% while combined with gentamicin or azithromycin. However, compound 1 showed the least efficacy in inhibiting bacterial biofilm production (about 60% in combination) than the other two compounds [88].

EPS consists of carbohydrate, nucleotide, and amino acid polymers are considered as major components of the biofilm matrix produced by *P. aeruginosa* [221]. Both compound 5 and 15 significantly reduced the level of biofilm carbohydrate, extracellular DNA, and proteins up to 90% while combined with gentamicin or azithromycin. Unfortunately, compound 1 was not adequate to reduce EPS compositions. They were also efficient to reduce essential virulence factors, the exoprotease activity (up to 93% inhibition), including LasB, rhamnolipid, and pyocyanin in combined treatment as well as inhibit motility movement compared to the antibiotic gentamicin or azithromycin alone [88,176]. This synergism was achieved by inhibiting the process of biofilm development, not by killing microbes. For *S. aureus* biofilm formation, compound 1 at a concentration of 50 μg/mL diminished biofilm thickness by about 45% on the polystyrene surface compared to the control set after 24 h exposure [139]. Reduction of biofilm thickness was dose-dependent manner, and the outcomes demonstrated that compound 1 has an important role in the inhibition of biofilm production in *S. aureus*.

### 5.2. Antiviral Effects

Since the last three decades, researchers have extensively studied the antiviral properties of *A. paniculata*. Although antiviral activity against a limited number of viruses viz. dengue virus serotype 1 [DENV-1] [182], herpes simplex virus type 1 [HSV-1] [181], influenza A virus [180], HIV [54,177,178,179], hepatitis B [186] and Hepatitis C [194] has been reported, their findings are very encouraging and significant considering the role of these viruses on human morbidity and mortality worldwide.

It is noteworthy that the formation of syncytia in co-culture of HIV-1 infected MOLT cell lines was significantly inhibited by the methanol extracts of *A. paniculata* [222]. The aqueous bark extracts of *A. paniculata* was investigated for HIV-1 protease inhibition activity, and this result supports an earlier report by Yao, et al. [223]. They reported positive results, but the extracts were less effective (29.6% and 26.3% inhibition at 250 µg/mL and 25 µg/mL, respectively) against HIV-1 protease [179]. Methanol extracts of *A. paniculata* showed antiviral effects against dengue virus (DENV) serotype-1 in vitro assay. After treating with the extracts, the viability of DENV-1 infected Vero E6 cells was 113 ± 4.65% with maximum non-toxic dose (0.050 mg/mL), and the percentage of inhibition was 75% [182]. Panraksa, et al. [93] evaluated andrographolide’s anti-viral activity against DENV serotype-2 in HepG2 and HeLa cell lines and DENV serotype-4 in one HepG2 cell line. They found a significant reduction of cellular infection and virus output levels in both cell lines, HepG2 (EC_50_ = 21.304 μM) and HeLa (EC_50_ = 22.739 μM) for DENV 2. The anti-viral activity of andrographolide was confined to a post-infection stage [93]. Andrographolide was more potent to inhibit DENV compared to the chikungunya virus (CHIKV). The CHICKV EC_50_ (77 μM) was about 3.5 fold higher than the DENV, comparable to two different DENV serotypes. In addition, andrographolide affected CHIKV replication [91]. Both cases in HepG2 and HeLa cell lines did not show any toxicity sign after treating with andrographolide at a maximum concentration of 100 μM for 24 h [91,93]. 

Reddy, et al. [177] have investigated several pure metabolites of *A. paniculata*, including bis-andrographolide ether, andrographolide, 14-deoxy-11,12-didehydroandrographolide, andrograpanin, 14-deoxyandrographolide, (±)-5-hydroxy-7,8-dimethoxyflavanone and 5-Hydroxy-7,8-dimethoxyflavone against HIV. Among these compounds, only andrographolide and 14-deoxy-11,12-didehydroandrographolide have demonstrated significant anti-HIV properties with (EC_50_ = 49.0 mg/mL) and (EC_50_ = 56.8 mg/mL). However, these outcomes were comparatively very less than the standard HIV treatment, azidothymidine (EC_50_ = 20 ng/mL) used for HIV. They used MT2 cells for the anti-HIV test and found a significant reduction of p24 antigen levels (doses used 5 to 100 mg/mL). The level of p24 antigen is one of the determinants of the anti-HIV effect since it is a viral protein that makes the viral capsid or core, and its expression is highest during the early phase of infection [224]. Andrographolide was also significantly effective to increase CD4^+^ lymphocytes in HIV patients. HIV RNA copy number was also decreased, but it was not significant [54].

Neoandrographolide, another most potent anti-HIV agent, have a unique C3-O-glucosyl moiety which plays a vital role in the inhibition of furin (IC_50_ = 53.5 µM). This activity is around 20-fold higher than the andrographolide (IC_50_ = 1.0 mM and K_i_ = 200 µM). Moreover, furin is a protease involved in the proteolysis of HIV envelop polyprotein gp120 prior to viral assembly. Gp120 helps viruses to attach to the specific cell surface receptor [225]. A derivative of andrographolide called dehydroandrographolide succinic acid monoester (DASM) was also experimented with in H9 (T-helper-cell line) cells and human peripheral blood mononuclear cells (PBMCs) and reported to have potential anti-HIV activity [178]. DAMS showed immense improved in vitro anti-HIV activity at the concentration of 50–200 (average, 108) µg/mL that was nontoxic to the H9 cells. It also demonstrated the inhibitory effect against HIV-1 and HIV-2 strains. They reported that the subtoxic concentration of DASM (200–400 µg/mL) partially interfered with HIV-induced cell fusion and binding of HIV virions to H9 cells. Probably, DASM might also be interfered with HIV replication by inhibiting HIV infected cell proliferation at another unidentified step(s).

Time- and dose-dependent hepatitis C virus (HCV) replication suppressive effect of andrographolide also reported earlier [194]. The researchers observed the synergistic effect of andrographolide in combination with IFN-α, an inhibitor targeting HCV NS3/4A protease or NS5B polymerase. The andrographolide’s effect was further linked to the up-regulation of heme oxygenase-1, which led to increased amounts of its metabolite (biliverdin) production that promoted the antiviral IFN responses inhibited NS3/4A protease activity which eventually suppressed HCV replication and showed anti-HCV activity [194]. In another study, dehydroandrographolide and andrographolide isolated from *A. paniculata* reported having hepatitis C virus (HBV) DNA replication suppressive activity with IC50 values of 22.58 54.07 μM and low SI values of 8.7 and 3.7 [186].

Another recent study conducted on human papillomavirus (HPV)-16 pseudovirus (HPV16PsV) to investigate the antiviral effect of andrographolide, it’s derivative- 14-deoxy-11,12-didehydroandrographolide and semi-synthetic analogue- 3,19-isopropylidene andrographolide (IPAD) [226]. They reported that all compounds inhibited HPV16PsV infection, of which 14-deoxy-11,12-didehydroandrographolide showed the highest potency. Additionally, only andrographolide suppressed the long control region (LCR) transcription activity of HPV16 in transiently transfected C33A cells [226].

Chen, et al. [180] reported considerable inhibitory activity (both in vitro and in vivo) of 14-ά-lipoyl andrographolide (AL-1), a synthetic derivative of andrographolide, against influenza A viruses H5N1, H9N2, and H1N1. It successfully prevents mortality in mice against H1N1 infection at the dose of 200 mg/kg/d, and 80% of mice survived at both dosages of 100 gm/kg/d and 200 gm/kg/d against H9N2 and H5N1 infections. It also demonstrated the most effective inhibition of viral adsorption onto red blood cells at the concentration of 5.3 to 16.8 mM, thereby inhibiting virus transmission to the uninfected cells. The similar result was observed by Aromdee, et al. [181] for 14-acetyl analogues of andrographolides (14-acetyl-3,9-isopropylideneandrographolide, 14-acetylandrographolide, 3,14,19-triacetylandrographolide) against HSV-1 in vitro. These three analogues were good for blocking viral entry in the pre-infection step. A cyclic dioxane analogue (3,9-isopropylideneandrographolide) was good for inhibiting viral replication at the post-infection level. However, andrographolide exhibited less effective inhibition activity against influenza A and HSV-1.

Prevention and management of coronavirus disease (COVID-19) have not yet successful since no specific preventive measurement and treatment available. Therefore, searching for potential bioactive compounds from natural sources is an ongoing investigation as medicinal plants possess a tremendous antiviral compound. In recent in silico studies, andrographolide [227], neoandrographolide [228], glycosides 5,4’-dihydroxy-7-O- β-D-pyran-glycuronate butyl ester [229] and glycoside 3-O-β-D-glucopyranosyl- andrographolide [229] have been reported to have a potential role in inhibiting the main protease of SARS-CoV-2, including NSP9, RNA-dependent RNA polymerase, and 6LU7. Andrographolide was docked successfully in the binding site of SARS-CoV-2 Mpro. Their findings revealed that the andrographolide molecule has good solubility, pharmacodynamics property and target accuracy [230]. In another very recent report also stated that andrographolide and its derivative-14-deoxy-11,12-didehydroandrographolide have strong binding affinities with targets. They can modulate the immune system by regulating chemokine signaling, Rap1 signaling, cytokine–cytokine receptor interaction, MAPK signaling, NF-kappa B signaling, RAS signaling, p53 signaling, HIF-1 signaling, and natural killer cell-mediated cytotoxicity [231]. A couple of in silico studies suggest strong interaction of andrographolide and its derivatives against COVID-19 associated target proteins and exhibited different immunoregulatory pathways; thus, these metabolites could be potential candidates for COVID-19 treatment, and further evaluation in vitro and in vivo would be worthy.

### 5.3. Antifungal Effects

*A. paniculata* crude extracts have been used for the treatment of fungal infections in folk medicines for centuries. The ethanol crude extract of the whole plant was reported to possess moderate antifungal activity against *A. oryzae* (60% inhibition) as well as *A. niger* (<60% inhibition) and *Penicillium* sp. (<40% inhibition) at 3% (*v*/*v*) concentration [232]. The hexane and methanol root extracts were evaluated for their antifungal activity against *A. niger* and *Penicillium chrysogenum*. Two concentrations (100 gm/mL and 200 mg/mL) of each extract were studied, which involved determining the inhibition zone diameter for a specific time. It was found that both extracts exhibited significant inhibition, 13 mm and 12 mm at 200 mg/mL concentration against *A. niger* and *Penicillium chrysogenum*, respectively. However, these inhibitions were less than the standard fluconazole, 17 mm and 16 mm at 100 µg/mL concentration, respectively [206].

Sule, et al. [183] first reported on the isolation of antifungal bioactive compounds from dichloromethane (DCM) and methanol extracts of *A. paniculata* whole plant. All the isolated bioactive, 3-O-β-D-glucosyl-14-deoxyandrographolide, 14-deoxyandrographolide and 14-deoxy-11,12-didehydroandrographolide, showed significant antifungal activity against *Microsporum canis*, *A. niger*, and *C. albicans*. MIC values for all antifungal compounds ranged from 50 to 150 μg/mL, and minimum fungicidal concentration (MFC) values ranged from 50 to 200 μg/mL. Among the isolated antifungal substances, 14-deoxyandrographolide exerted the lowest MIC (50 μg/mL) and MFC (50 μg/mL) against *M. canis*, which indicates the most potent antifungal activity. It is noted that no anti-fungal activity was reported against *T. mentagrophytes* and *T. rubrum* at 250 μg/mL. Even though different extracts of *A. paniculata* reported to have potential anti-fungal activity, the mode of actions yet underreport.

### 5.4. Anti-Parasitic Effects

*A. paniculata* and its bioactive compounds have been tested and found extensive anti-parasitic activity against various parasites, such as *Ascaris lumbricoidis*, *Plasmodium falciparum*, *P. berghei*, *Trypanosoma cruzi*, etc. The extract and fractions reduced parasitaemia level in *Mastomys natalensis* while used in a dose-dependent manner [184]. Misra, et al. [184] have also studied the anti-malarial activity of the four diterpenes-andrographolide, neoandrographolide, deoxyandrographolide and andrographolide-isolated from *A. paniculata* and revealed that neoandrographolide (2.5 mg/kg BW) exhibit the highest activity when administered by gastric lavage than other diterpenes.

The fractions of *A. paniculata* also possessed significant anti-malarial activity [185]. The methanol extract was found to have complete inhibition at a concentration of 2.5 mg/mL by 48 h. Chloroform extract also achieved the same effect by 24 h at only 0.05 mg/mL concentration [233]. The isolated andrographolide also exhibited substantial anti-malarial activity against the MRC-pf-303 strain of *P. falciparum* and particularly inhibited the parasites at the ring stage [234]. Consequently, the methanol fractions soluble in chloroform were evaluated as significant inhibition of parasitaemia (74%) at the concentration of 1 mg/mL. Additionally, andrographolide showed the highest (53.9%) inhibition of parasitaemia level [235].

It has been found that the alcohol rhizome extract possessed significant in vitro activity against *A. lumbricoides* [236]. Two reviews were also reported *A. paniculata* rhizome exhibited extensive activity against *A. lumbricoides* [35,36]. Dutta and Sukul [237] have studied in vitro anti-filarial activity of leaves decoctions of *A. paniculata* against *Dipetalonema reconditum* and found the decoctions kill microfilaria in 40 min. In vivo study revealed that more than 85% of microfilaria in the blood reduced after three subcutaneous injections of the extract into infected dogs at 0.06 mL/kg BW.

## 6. Controlled Clinical Trials of *A. paniculata* Treatment: A Systematic Evaluation

*A. paniculata* and/or its bioactive compounds have been used to treat patients with uncomplicated upper respiratory tract infections (URTIs), including common cold, rhinitis, nasopharyngitis, pharyngitis, and pharyngotonsillitis. The surrounding bacteria and viruses are the usual source of infection for the URTIs. In the treatment of URTIs, pills (made by whole powdered plant and water) and tablets (water extract of the plant) have been used, and the cure rates are 88% and 61%, respectively. The therapeutic effectiveness differed mainly due to the preparation method and duration of treatment [36]. Several randomized, double-blind, placebo-controlled trials proved the efficacy of *A. paniculata* standardized extracts and/or andrographolide. Some other bioactive compounds treat various infectious diseases associated with cold symptoms and infections caused by virus-like influenza [55,56,57,58,59,60,238,239,240,241,242].

**Figure 6:**
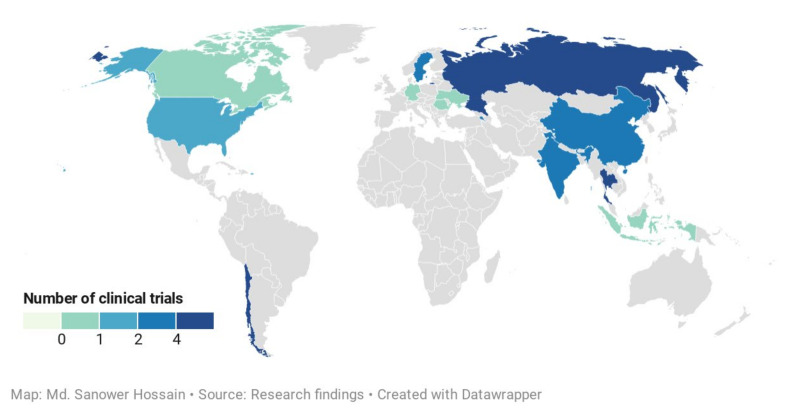
Global distribution of controlled clinical trials of *Andrographis paniculata*. (This map indicates the location of clinical studies conducted and color intensity indicated the number of studies frequencies. One trial conducted by Sandborn, et al. [63] covered five countries: the USA, Canada, Germany, Romania, and Ukraine).

Our systemic investigation revealed a total of 41 individual clinical trials after removing the duplicates from three different databases. After critical evaluation, 23 individual trials (*n* = 2760) were finally selected for this study which was distributed in 13 different countries (Figure 6). These included 11 controlled clinical trials of the treatment of uncomplicated URTIs (eight studies) [56,58,59,60,238,239,240,242], viral infections (three studies)-influenza (two studies) [55,57] and HIV (1 studies) [54], and one trial focused on the immunity enhancement [241] that prevent the occurrence of the common cold in a rural school-going healthy student (Table 3). The remaining controlled clinical trials are 11 that covered the *A. paniculata* treatment on the healthy volunteers (three studies)-physiological effects [12], semen quality and fertility [243], pharmacokinetics [244], two studies each for ulcerative colitis [62,63], arthritis pain [66,245], and other studies include one trial each of Familial Mediterranean Fever (FMF) [246], hypertriglyceridemia [64], fatigue [67], type 2 diabetes mellitus [61].

**Figure 7:**
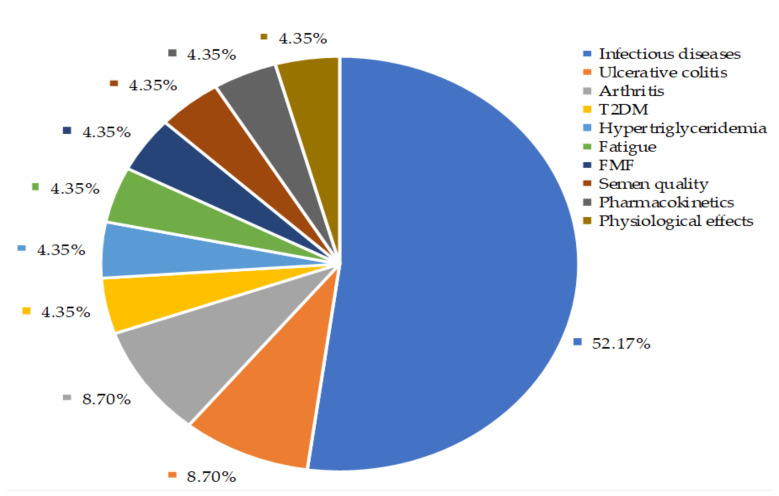
Controlled clinical trials of *Andrographis paniculata*. Infectious diseases include uncomplicated upper respiratory infections (UTRI) (i.e., common cold, rhinitis, nasopharyngitis, pharyngitis, and pharyngotonsillitis) and viral diseases (i.e., influenza, HIV). FMF: Familial Mediterranean Fever; T2DM: Type 2 diabetes mellitus.

Our findings depicted that about 52% of controlled clinical trials were conducted to investigate the *A. paniculata* extracts and their bioactive compounds effects on the disappearance of common cold, uncomplicated UTRIs symptoms, as well as recovery from viral infections including influenza and HIV (Figure 7). Among these studies, eight were randomized, double-blind, placebo-controlled studies. The characteristics of these studies and treatment outcomes are shown in Table 3. The details of *A. paniculata* treatment outcomes on the other health conditions and physiological effects on the healthy volunteer (HV) are given in Supplementary Table S3.

### 6.1. Evaluation of A. paniculata Efficacy Against Infections

Eleven randomized, and 1 non-randomized clinical trials (*n* = 2008) were met inclusion criteria. The compliance rate of this study was 95.97%. Of these, nine were double-blind, of which eight were placebo-controlled. Others were either simple randomized control or randomized parallel-group or randomized controlled open-label. Melchior, et al. [240] and Kulichenko, et al. [57] included one pilot trial and one main trial in one article. Therefore, we split their findings and presented them separately in Table 3.

A comparative, randomized, double-blind study [60] had been done on 152 Thai patients with pharyngotonsillitis to investigate the efficacy of *A. paniculata* extracts using either paracetamol (3.9 g/day) or encapsulated *A. paniculata* dried leaves for low dose group (LDG) (3 g/day) or high dose group (HDG) (6 g/day) for seven days. There was no significant difference in efficacy of relieving fever (*p* = 0.16) and sore throat (*p* = 0.49) among three groups on day 7. Most of Paracetamol and HDG stopped taking medication on day three because their symptoms had disappeared. However, LDG patients discontinued taking medications due to persistent adverse side effects or worsening symptoms. The incident of mild or self-limiting side effects (i.e., nausea, vomiting, abdominal discomfort, dizziness, drowsiness, and malaise) was not statistically significant among the three groups (*p* = 0.8), and patients were almost equally satisfied with paracetamol and a high dose of *A. paniculata* treatment. The findings indicated that daily 6 g of *A. paniculata* dried leaves extracts that contain at least 6% of andrographolide can be replaced by paracetamol treatment as a standard treatment for the patients with symptoms of pharyngotonsillitis.

In a placebo-controlled study conducted by Caceres, Hancke, Burgos and Wikman [241], Kan Jang tablets, a standardized *A. paniculata* extract, had been administrated to 107 healthy students in a rural school at a dose of two tablets (200 mg) per day for three months to evaluate the efficacy of Kan Jang to prevent common colds. The common colds were successfully prevented; 2.1-fold higher prevention in the Kan Jang group compared to the placebo group. The incidence of common colds was 30% (16 out of 54) and 62% (33 out of 53) in the Kan Jang group and placebo group, respectively. Melchior, et al. [58] conducted another similar study on 50 volunteers suffering from common colds and sinusitis using Kan Jang tablets for five days. Both subjective symptoms and duration of the symptoms were significantly reduced; 68% of patients recovered entirely in the Kan Jang group. In contrast, only 36% of patients recovered in the placebo group.

Another randomized, double-blind placebo-controlled study on 158 adult patients of both sexes used *A. paniculata* dried extract SHA-10 for five days to investigate the efficacy in plummeting the prevalence and intensity of sign and symptoms of common colds. The extract (1.2 g/day) showed the ability to reduce the intensity of the symptoms of tiredness, sleeplessness, sore throat, and nasal secretion compared to the placebo group. The intensity of symptoms started to decrease from the second day. All symptoms disappeared on day four without showing any adverse effect [242]. In similar two other Swedenian studies, the Kan Jang, a standardized extract of *A. paniculata* in a fixed combination with *Eleutherococcus senticosus*, has been used three times daily for 3–8 days. This study evaluated the efficacy in the treatment of uncomplicated URTIs on 46 and 179 patients, respectively. The treatment group showed highly significant improvement compared to the placebo group in terms of both the total symptom score (*p* < 0.0006) and total diagnosis score (*p* < 0.003). In addition, relief of throat symptom was highly significant in both studies [240].

A three-arm clinical study has been carried out on 130 children (ages 4–11 years) over ten days to evaluate the effects of Kan Jang on uncomplicated respiratory disease-common colds. In the three groups, the control group treated with standard common cold treatment and the other two groups adjuvant and adjuvant control group treated with Kan Jang tablets and Immunal (a preparation of *Echinacea purpurea* L. extract) concomitant to standard treatment, respectively. The Kan Jang showed more significant effects by the rapid recovery process. Using a less standard medication, resulting in the symptoms, particularly nasal secretion and congestion, was less severe at an early stage of uncomplicated common colds in the Kan Jang treatment group. However, Immunal did not exhibit the same efficacy. The use of Kan Jang as an alternative medication was found to be safe and efficacious in treating uncomplicated upper respiratory tract diseases [238].

A randomized, controlled clinical study has also been conducted to investigate Kan Jang’s effects in treating influenza viral infection on 540 patients. In (same as before) group, Kan Jang treated patients recovered very quickly from infections, and Kan Jang reduced the risk of post-influenza complications. Moreover, patients tolerated the Kan Jang very nicely [57]. In addition, the addition of *A. paniculata* to paracetamol in influenza patients could further reduce the severity of influenza symptoms compared to the control group who was given paracetamol alone [55].

Existing clinical studies showed that *A paniculata* has a clinical effect on infectious diseases and influences several other diseases. *A. paniculata* can decrease fasting and postprandial glucose and decrease body mass index in diabetes mellitus patients. *A. paniculata* also influences mild to moderately active ulcerative patient. Administration of 1800 mg showed a significant clinical response (rectal bleeding) compared to placebo (*p* = 0.0183) [61].

### 6.2. Evaluation of Safety of A. paniculata Treatment

The incidents of the adverse effect of treatment with the natural product are sporadic. To our best knowledge, there was no such severe adverse event reported for *A. paniculata* treatment to the drug safety body. Our critical observations revealed that *A. paniculata* treatment showed a mild adverse event in some cases. Out of 14 reports (Table 4), six reports stated (*n* = 579) that they did not observe any adverse effects (or report not provided). These studies cover only 1991–2010. Our systematic investigation did not reveal any clinical studies conducted from 2010 to now. However, several clinical studies conducted using *A. paniculata* extracts or pure compounds on the healthy volunteer to check semen quality [243], pharmacokinetics and tolerance ability [244], ulcerative colitis [62,63], arthritis [66,245], fatigue [67], Familial Mediterranean Fever [246], hypertriglyceridemia [64], and type 2 diabetes mellitus [61] (Supplementary Table S3).

Saxena, et al. [59] (*n* = 223) reported mild adverse effect (2.73%): vomiting (1 case), epistaxis (1 case), Urticaria (1 case) and diarrhoea (3 case). Except for vomiting (patient in the treatment group) and urticaria, all other effects stopped spontaneously without any medication. Minimal and self-limiting side effects (*n* = 152) (i.e., nausea, vomiting, abdominal discomfort, dizziness, drowsiness, and malaise) were found about 20% in treatment (LDG & HDG) and paracetamol groups (9–11 cases) [60]. These are pervasive mild effects that usually recovered shortly without any medications. Three cases out of 200 subjects were also reported mild side effects: increase in nasal discharge and epigastric pain (1), nose blocked (1), and severe headache (1) for the treatment of *A. paniculata* fixed combination Kan Jang in URTIs and sinusitis [239]. For viral infections, the treatment group (Kan Jang) experienced dry cough, rhinitis, and pain in the throat (22 cases out of 540). Control group received antiviral agent amantadine which showed significantly (*p* < 0.01) higher (67.8% cases) influenza complications compared to treatment group (30.1% cases) [57]. For main trials, influenza complications were found in 31.43% of *A. paniculata* treated patients and 70.97% of standard medicine (amantadine) treated patients (*p* < 0.01). one HIV positive experience an anaphylactic reaction in phase I clinical trials [54]. All but one (92%) reported at least one adverse event during the study. About 75% reported an adverse event by the healthy volunteer. All conditions were returned to normal by week 9 [54]. *A. paniculata* standardized extract treatment of uncomplicated acute URTI patient experienced unpleasant sensations in the chest and intensified headache (1 case out of 180) [240], and for common cold and sinusitis, two patients out of 50 experienced urticaria [58].

Clinical studies other than respiratory infections also reported no adverse effects or mild effects. Only two patients (*n* = 180) with mild-to-moderate ulcerative colitis experienced severe adverse effects [63]. However, clinical response was significantly higher in the *A. paniculata* group than in the placebo (*p* = 0.0183). The best efficacy was observed with the HDG of *A. paniculata*. In another clinical trial with ulcerative colitis (*n* = 108), both *A. paniculata* extract-treated group and control group experiences several side effects, including aphthous ulcer (1), WBC decrease (1), abdominal pain (1), blood in the stool (1), fever (1), elevated glucose (1), rash (1) [treatment group]; blood in the urine (2), elevated CRP (1), WBC decrease (1), blood in the urine (2), fever (1), WBC decrease (1), abdominal pain (1), dry mouth (1), oedema lower extremity (1) cough (2), diarrhoea (2), dizziness and nausea (1), WBC elevated in urine (1) and other [control group], where comparatively treatment groups showed less adverse effects [62]. This indicated that the drug tolerance capacity of ulcerative patients is less regardless type of drugs, and they should be taken precaution measurement before taking any medicine, including *A. paniculata*.

Considering the type of adverse events and frequency and overall efficacy in treating subjective symptoms of common cold or URTIs either alone or in combination with *Acanthopanax senticosus*, *A. paniculata* might be safe for both adults and children. However, the treatment must need to follow the prescribed regime and daily recommended dose. Based on the findings of this study, 90–150 mg andrographolide daily could be recommended as a safe dose for the treatment of URTIs and other similar complications. Additionally, URTIs patients with ulcerative colitis should take extensive precaution taking any medications, and they are not recommended or encouraged for self-medication of any herbal drugs, including *A. paniculata*.

## 7. Methodology

The information related to this article was systematically collected from worldwide accepted scientific databases including PubMed (http://www.ncbi.nlm.nih.gov/pubmed (accessed on 27 March 2021)), ScienceDirect (http://www.sciencedirect.com/ (accessed on 27 March 2021)), Scopus (http://www.scopus.com/ (accessed on 27 March 2021)), Web of Science (https://apps.webofknowledge.com/ (accessed on 27 March 2021)), Springer Nature (http://link.springer.com/ (accessed on 27 March 2021)), Wiley Online Library (http://onlinelibrary.wiley.com/ (accessed on 27 March 2021)), and advanced search in Google Scholar (http://scholar.google.com.my/ (accessed on 27 March 2021)), as well as recognized books, abstract, and thesis/dissertation using the keywords “*Andrographis paniculata*”, “antimicrobial”, “anti-bacterial” “antiviral” “antifungal”. In the aforementioned databases, other relevant papers from the list of references of all available articles were searched. For searching the controlled clinical trials, the following keywords: “antiparasitic”, “clinical trials”, “controlled clinical trials”, and “randomized clinical trials” were used in PubMed, Scopus, and web of science. Controlled clinical trials were systematically screened and selected for further evaluation of their outcomes in this study. The studies were selected for this review (clinical section) if they were controlled clinical trials dealing with *A. paniculata* to treat infections incredibly uncomplicated upper respiratory tract infection. Used of *A. paniculata* for other health conditions and healthy volunteers were also selected in this study. English language only restriction was imposed.

## 8. Conclusions and Recommendations

Human invasive microbes are growing resistant to the available antibiotics for many reasons. As *A. paniculata* works on immune regulation, there are fewer chances of drug-resistance occurrence. Even though *A. paniculata* has a potential antimicrobial activity, the detailed study regarding the mode of actions, effects concerning the available antimicrobial agents and specific administration route as well as schedule remain to be explored. The active constituents of *A. paniculata* could be a potential source of antimicrobial agent, and exploring the therapeutic potentiality of them based on the clinical implications are worthwhile. We have explored substantial antimicrobial agents in *A. paniculata* (Table 1); however, very little is still known about their molecular mechanisms in response to microbes or host-infected cells. A majority of metabolites are not investigated to identify the molecular target to understand the drug-target-disease network. In addition, our checklist of secondary metabolites of *A. paniculata* (Supplementary Table S2) can be used for further exploration of their effectiveness because about 44% of metabolites are yet to be evaluated.

The outcomes from clinical effects suggest that *A. paniculata* offers a promising treatment for alleviating symptoms of infections caused by bacteria or virus that appeared in the URTIs. Nevertheless, a few common mild adverse events were reported from both short term and long term treatments. Therefore, self-medication using *A. paniculata* should be cautious to avoid potential adverse effects such as vomiting, diarrhea etc. If we consider the overall efficacy of *A. paniculata* treatment, it would be a worthy consideration as a natural product treatment option for acute URTIs as currently, there is a lack of compelling therapeutic opportunity for IDs.

In some cases, pure compounds from medicinal plants, such as aristolochic acids, possess severe side effects like kidney failure and urinary tract cancers [247]. Therefore, moving forward, the further requires us to take a more comprehensive approach to harness the true potential of *A. paniculata* for IDs fully. Different disease conditions have diverse responses to the drugs; therefore, to obtain a complete picture of the drug-target-disease network, elucidating each secondary metabolite’s mechanism(s) is crucial. Andrographolide has the potential to target multiple sites since it has shown significant efficacy against different disease conditions. Therefore, this natural product could be considered as a potential candidate for polypharmacology. We would obtain the full advantage of using andrographolide for therapeutics in the near future.

## Figures and Tables

**Table 3:** Study characteristics and treatment outcomes of controlled clinical trials of *Andrographis paniculata* in treating uncomplicated upper respiratory tract infections and viral diseases.

Study ID; Year; Country	Study Design	Gender & Age	Recruitment(n)/Analyzed(n)	Diagnosis	Study Medications	Daily Dosage (Duration)	Active Ingredients	Salient Outcomes
Thamlikitkul, et al. [60]; 1991; Thailand	R, DB	G: M&F A: 12 y or older	n = 152/142 CR = (93.43%) AP (LDG) = 48/46 AP (HDG) = 51/47 Paracetamol = 53/49 BCS = NSD (*p* > 0.05)	Pharyngotons- illitis	AP dried leaves extract (250 or 500 mg/capsule) and paracetamol (325 mg/capsule)	3 capsules 4 xD (7 d)	6% of AND	There was NSD in the efficacy of relieving fever (*p* = 0.16) and sore throat (*p* = 0.49) among 3 groups on day 7. The majority of the Paracetamol and AP (HDG) group patients stopped taking medication on the 3rd day due to relief of symptoms.
Hancke, et al. [56]; 1995; Chile	R, DBPC	G: M&F A: 18–60 y	n = 59/59 CR = 100% AP = 33/33 P = 28/28 BCS = NSD (*p* > 0.05)	Common cold	Monodrug Kan Jang: AP dried extract (100 mg/tablet)	1.2 g daily (4 d)	4% of AND	AP extract attenuates the signs of a common cold significantly at day 4 after treatment which is not observed with the placebo (*p* < 0.05).
Caceres, et al. [241]; 1997; Chile	R, DBPC	G: M&F A: ~18 y	n = 107/107 CR = 100% AP = 54/54 P = 53/53	Healthy volunteer *	Monodrug Kan Jang: AP dried extract (100 mg/tablet)	Daily 2 tablets, 5 d/w (3 m)	5.6% of AND	After the third month of treatment, a significant decrease in common colds in the AP group was observed compared to the P group (*p* < 0.05).
Melchior, et al. [58]; 1997; Sweden	R, DBPC	G: M&F A: 18–55 y	n = 50/50 CR = 100% AP = 25/25 P = 25/25 BCS = NSD (*p* > 0.05)	Common cold sand sinusitis	AP leaves hydroalcoholic extract (85 mg/tablet)	4 tablets 3 xD (5 d)	AND & DAND	The total recovery rate was 67.5% and 36% in Kan Jang and placebo group, respectively (*p* < 0.046).
Melchior, et al. [240]; 2000; Sweden	R, DBPC	G: M&F A: 18–55 y	n = 47/46 CR = 97.87% AP = 23/23 P = 24/23 BCS = NSD (*p* > 0.05)	Uncomplicated acute URTI	Combination of APE and AS extract (85 mg/tablet)	3 tablets 4 xD (5–6 d)	5.25 mg AND & DAND; 9.7 mg per tablet EB and EE	Much improvement in the patient’s overall symptoms cores in TrG was observed compared to P (*p* = 0.08).
Melchior, et al. [240]; 2000; Russia	R, DBPC	G: M&F A: 18–55 y	n = 180/179 CR = 99.44% AP = 90/89 P = 90/90 BCS = NSD (*p* > 0.05)	Uncomplicated acute URTI	Combination of APE and AS extract (85 mg/tablet)	3 tablets 4 xD (5–6 d)	55.25 mg AND & DAND; 9.7 mg per tablet EB and EE	The difference between TrG and P groups was significant for total diagnosis score (*p* = 0.003) and total symptom score (*p* = 0.0006).
Caceres, et al. [242]; 1999; Chile	R, DBPC	G: M&F A: 25–50 y	n = 208/158 CR = 87.78% AP = 102/79 P = 106/79 BCS = NSD (*p* > 0.05)	Common colds	APE (100 mg/tablet)	4 tablets 3 xD (5 d)	5% of total AND & DAND	On day 4 of treatment, the decrease in the intensity and duration of symptoms was highly significant between TrG and P groups (*p* < 0.001).
Gabrielian, et al. [239]; 2002; Armenia	PG, DBPC	G: M&F A: 15–64 y	n = 200/185 CR = 95.45% AP = 100/95P = 100/90 BCS = NSD (*p* > 0.05)	Acute URTIs and sinusitis	Combination of APE and AS extract (85 mg/tablet)	4 tablets 3 xD (5 d)	5 mg AND & 10 mg per tablet	Headache, nasal, sore and dry throat, and general malaise showed the most significant improvement (*p* < 0.001), while cough and eye symptoms did not differ significantly between the groups.
Spasov, et al. [238]; 2004; Russia	RC3PG	G: M&F A: 4–11 y	n = 133/133 CR = 100% Group A (AP) = 53/53 Group B = 41/41 Group C = 39/39 BCS = SD; NSD (BP, *p* > 0.05)	Uncomplicated URTI	Combination of APE and AS extract (85 mg/tablet); Immunal drops: contain EP and ethanol (4:1)	A: 2 tablets 3 xD (10 d); B: 10 drops 3 xD (10 d);C: 500 mg paracetamol 3 xD (10 d) and others^ℓ^	5.25 mg AND & DAND; 9.7 mg per tablet EB and EE	Compared with group B (Immunal), the AP extract group considerably improved the development of the disease and intensified children’s recovery with common colds.
Saxena, et al. [59]; 2010; India	R, DBPC	G: M&F A: 18–60 y	n = 223/220 CR = 98.65% AP = 112/112P = 111/108 BCS = NSD (*p* > 0.05)	Uncomplicated URTIs	KalmCold™ (dried AP leaves extract; formulated by mixing methanol and water extract) (100 mg/capsule)	1 capsule 2 xD (5 d)	AND, IAND, NAND, AGN, DDHAND, SCF & MWN	The intervention group showed 2.1 folds higher improvement in reducing URTI symptoms than the placebo group (*p* ≤ 0.05).
Kulichenko, et al. [57]; 2003; Russia	RPG	G: M&F A: 19–63 y	n = 540/540 CR = 100% G (AP) = 71/71 Crt = 469/469 BCS = NSD (*p* > 0.05)	Influenza	AP: Combination of APE (88.8 mg) and AS (10 mg) extract (100 mg/tablet) Ctr: STD^€^	AP: 2 tablets 3 xD (3–5 d); Ctr: STD^€^	AP: 5.25 mg AND & DAND; 9.7 mg per tablet EB and EE;Ctr: amantadine	Kan Jang treated patients recovered (69.9%) very quickly and reduced the risk of post-influenza complications, while Ctr group recovered 32.2% only (*p* < 0.001).
Kulichenko, et al. [57]; 2003; Russia	SRC	G: M&F A: 20–63 y	n = 66/66 CR = 100% AP = 35/35 Ctr = 31/31 BCS = NSD (*p* > 0.05)	Influenza	AP: Combination of APE (88.8 mg) and AS (10 mg) extract (100 mg/tablet) Ctr: STD^€^	AP: 3 tablets 3 xD (5 d); Ctr: Standard therapy	AP: 5.25 mg/tablet AND & DAND, EB and EE; Ctr: amantadine	AP extract significantly reduced clinical symptoms and sped up patients’ recovery, and significantly (*p* < 0.001) decreased the number of days off work and the number of cases with post-influenza complications.
Calabrese, et al. [54]; 2000; USA	NRCT	G: M&F A: 18 y and above	n = 18/17 CR = 94.44% AP = 13/13HV = 5/4 BCS = NSD (*p* > 0.05)	HIV	AND	5, 10, 20 mg/kg BW 3 xD first 3 w, second 3 w and last 3 w, respectively	AND	A dose of 10 mg/kg andrographolide administration significantly (*p* = 0.002) increased the mean CD4+ lymphocyte count (405 cells/mm3 to 501 cells/mm3) in HIV patients.
Chuthaputti, et al. [55]; 2007; Thailand	RCOL	G: M&F A: 3–15 y	n = 25/25 CR = 100% AP = 15/15 Ctr = 10/10 BCS = NSD (*p* > 0.05)	Influenza	AP: Paracetamol and AP aerial part extract (400 mg/capsule); Ctr: Paracetamol (500 mg/tablet)	AP: 4 capsules 4 xD; Ctr: 2 tablets 4 xD (7 d)	9% of AND, Paracetamol	The severity of cough, fatigue, and overall symptoms of the TrG were significantly lower than the Ctr group from Day 4 onwards.

* To improve immunity and diminish the occurrence of common colds among the rural school children; A: Age; AGN: Andrograpanin; AND: Andrographolide; AP: *Andrographis paniculata*; APE: AP extract; AS: Acanthopanax senticosus; AS: Acanthopanax senticosus; BCS: Baseline characteristics; BP: Bacterial population; BW: Body weight; CR: Compliance rate; CrG: Control group; Ctr: Controlled; d: Day; DAND: Deoxyandrographolide; DAS: Dehydroandrographolide succinate; DBPC: Double-blind, placebo-controlled; DDHAND: 14-deoxy-11,12- didehydroandrographolide; EB: Eleutheroside B; EE: Eleutheroside E; ES: Echinacea purpurea; F: Female; G: Gender; Gr: Group; FMF: Familial Mediterranean Fever, HDG: High dose group; HV: Healthy volunteer; ImmunoGuard®: 370 mg, containing a fixed combination of AP special extract (50 mg) standardized for the content of andrographolide(3-[2-[-decahydro-6-hydroxy-5-(hydroxymethyl)-5,8a-dimethyl-2-methylene 1-naphthalenyl] ethyldiene] dihydro-4-hydroxy-2(3H)-furanone)–4 mg, *Eleutherococcus senticosus* special extract (10 mg) standardized for the content of Eleutherosid E (>0.8 mg), Schizandra chinensis special extract (100 mg) standardized for the content of Schisandrins (>0.8 mg), Glycyrrhiza glabra L extract (10 mg) standardized for the content of Glycyrrhizin (>0.6 mg), 190.3 mg microcristalline cellulose, 7.4 mg Syloid FP, 1.8 mg magnesium stearate, 0.8 mg shellac, 0.1 mg olive oil, and 0.07 mg Macrogol; IAND: Isoandrographolide; IV: Intravenous administration; LDG: Low dose group; M: Male; Mix: Mixture; m: Month; MWN: 7-O-methylwogonin; n: Sample size; NAND: Neoandrographolide; NRCT: Non-Randomized Controlled Trial; NSD: No significant difference; PG: Parallel-group; R: Randomized; R3WC: Randomized, three-way crossover; RC3PG: Randomized Controlled three parallel group; RCOL: randomized controlled open-label; RPG: Randomized Parallel-group; SCF: skullcapflavone I; Rt: Recruitment; SRC: Simple randomization via computer; STD: Standard treatment; STD^€^: mainly amantadine (antiviral agent) with ascorbic acid as an adjuvant. Each tablet contains 50 mg amantadine. 1st day– 2 tablets 3 xD, 2nd & 3rd day– 2 tablets 2 xD, 4th day– 2 tablets 1 xD; ℓ: standard treatment of lavish warm drinks, throat gargles, antiseptic nose drops, and paracetamol; TD: Treatment duration; TrG: Treatment group; URTIs: Upper respiratory tract infections; VO: *Valeriana officinalis*; w: Weeks; xD: times daily; Y: Year.

**Table 4:** Adverse events reported in the treatment of human subjects using *A. paniculata* for uncomplicated upper respiratory tract infections and viral diseases.

Reference	Study Design	Recruitment(n)/Analyzed(n)	Diagnosis	Adverse Effects (Cases)	
				Treatment	Placebo
Thamlikitkul, et al. [60]	R, DB	n = 152/142	Pharyngotonsillitis	Minimal and self-limiting side effects (i.e., nausea, vomiting, abdominal discomfort, dizziness, drowsiness, and malaise) were found about 20% in treatment (LDG & HDG) and paracetamol groups (9–11).	No placebo groups.
Hancke, et al. [56]	R, DBPC	n = 59/59	Common cold	No adverse event reported	No adverse event reported.
Caceres, et al. [241]	R, DBPC	n = 107/107	HV *	Not reported adverse effect information	Not reported adverse effect information
Melchior, et al. [58]	R, DBPC	n = 50/50	Common colds and sinusitis	Urticaria (2)	No adverse event reported.
Melchior, et al. [240]	R, DBPC	n = 47/46	Uncomplicated acute URTI	No adverse event information reported	No adverse event information reported
Melchior, et al. [240]	R, DBPC	n = 180/179	Uncomplicated acute URTI	Unpleasant sensations in the chest and intensified headache (1)	No adverse event reported.
Caceres, et al. [242]	R, DBPC	n = 208/158	Common colds	No adverse events were observed	No adverse event reported.
Gabrielian, et al. [239]	PG, DBPC	n = 200/185	URTIs and sinusitis	Increase in nasal discharge and epigastric pain (1), nose blocked (1), and severe headache (1). They were excluded from the analysis data.	No adverse event reported.
Spasov, et al. [238]	RC3PG	n = 133/133	Uncomplicated URTI	No side effects were observed	No placebo group
Saxena, et al. [59]	R, DBPC	n = 223/220	URTIs	Mild adverse effect; vomiting (1), epistaxis (1), Urticaria (1) and diarrhoea (3). Except for vomiting (patient in AP group) and urticaria, all other effects stopped spontaneously without any medication.	No adverse event reported.
Kulichenko, et al. [57]	RPG	n = 540/540	Influenza	AP group: Dry cough, rhinitis, and pain in the throat (22). Influenza complications were found in 30.1% of the AP group and 67.8% of the Ctr group (*p* < 0.01).	No placebo group
Kulichenko, et al. [57]	SRC	n = 66/66	Influenza	Influenza complications were found in 31.43% of AP-treated patients and 70.97% of STD^€^-treated patients (*p* < 0.01).	No placebo group
Calabrese, et al. [54]	NRCT	n = 18/17	HIV	Anaphylactic reaction (1). All but one (92%) reported at least one adverse event during the study. ¾ reported an adverse event by the healthy volunteer. All conditions were returned to normal by week 9.	No placebo group
Chuthaputti, et al. [55]	RCOL	n = 25/25	Influenza	No adverse event information reported.	No placebo group

* To improve immunity and diminish the occurrence of common colds among the rural school children; AP: *Andrographis paniculata*; DBPC: Double-blind, placebo-controlled; HDG: High dose group; HV: Healthy volunteer; LDG: Low dose group; NRCT: Non-Randomized Controlled Trial; PG: Parallel group; R: Randomized; R3WC: Randomized, three-way crossover; RC3PG: Randomized Controlled three parallel-group; RCOL: randomized controlled open-label; RPG: Randomized Parallel-group; SRC: Simple randomization via computer; STD^€^: mainly amantadine (antiviral agent) with ascorbic acid as an adjuvant. Each tablet contains 50 mg amantadine; URTIs: Upper respiratory tract infections.

## Data Availability

Data is contained within the article or Supplementary Material.

## Supplementary Materials

The following are available online at https://www.mdpi.com/article/10.3390/life11040348/s1, Table S1. Invasive test microbes for the evaluation of antimicrobial efficacy of different extracts of *Andrographis paniculata* and its isolated metabolites, Table S2. Isolated pure metabolites of *Andrographis paniculata* and the part used, Table S3. Study characteristics and intervention outcomes of clinical trials of *Andrographis paniculata* for healthy volunteers and other health complications included familial Mediterranean fever, diabetes mellitus, hypertriglycemia, ulcerative colitis, fatigue and arthritis pain.
