# Supplementary material for: Andrographis paniculata (Burm. f.) Wall. ex Nees: An Updated Review of Phytochemistry, Antimicrobial Pharmacology, and Clinical Safety and Efficacy
Source: Life (Basel). 2021 Apr 16;11(4):348. doi: 10.3390/life11040348 (PMC8072717; doi:10.3390/life11040348)
Supplement: Supplementary file 1 [file life-11-00348-s001.zip › life-1140970/Supplementary Table S1.docx]

**Table S1.** Invasive test microbes for the evaluation of antimicrobial efficacy of different extracts of *Andrographis paniculata* and its isolated metabolites.

| **SL#** | **Species** | **Gram Staining** | **Mode of Infection/ Transmission** | **Diseases/Infections** | **Infected Organism** | **Ref** |
| --- | --- | --- | --- | --- | --- | --- |
|  | Bacteria |  |  |  |  |  |
|  | *Aeromonas hydrophila* | Negative | During direct ingestion or drinking of contaminated water and food, oral-facial transmission | Diarrhoea, cellulitis, wound infection, hepatobiliary, septicemia | Human, fish, amphibians, reptiles, birds | [1] |
|  | *Bacillus anthracis* | Positive | Through facial or oral | Cutaneous anthrax, Pulmonary anthrax, Gastrointestinal anthrax | Human | [2,3] |
|  | *Bacillus licheniformis* | Positive | Silage, water, food stuff.  Raw milk, industrially produced baby food | No adverse health effects | Cow, human | [4] |
|  | *Bacillus pumilus* | Positive | Food poisoning | Central venous catheter | Human | [5] |
|  | *Bacillus subtilis* | Positive |  | No sign of pathogenicity |  | [5-11] |
|  | *Bordetella pertussis* | Negative | These bacteria attach to the cilia (tiny, hair-like extensions) that line part of the upper respiratory system. The bacteria release toxins (poisons), which damage the cilia and cause airways to swell. | Whooping cough, Bacterial pneumonia | Human | [12] |
|  | *Edwardsiella tarda* | Negative | Through the faeces | (fish pathogen) Edwardsiellosis | Fish, amphibians, reptiles, mammals, human | [1] |
|  | *Enterobacter cloacae* | Negative | Infected through the skin, gastrointestinal tract, urinary tract or derived externally due to the ubiquitous nature | Nosocomial infection, bacteremia, lower respiratory tract infections, skin and soft tissue infections, urinary tract infections, endocarditis, intra-abdominal infections, septic arthritis, osteomyelitis, and ophthalmic infections | Human | [8] |
|  | *Enterococcus faecalis* | Positive | Infected through the faecal matter | Nosocomial infection, (less common) meningitis, hematogenous, osteomyelitis, septic arthritis, and (very rarely) pneumonia | Human | [2,8] |
|  | *Escherichia coli* | Negative | Contaminated water or food especially raw vegetables and undercooked ground beef | Urinary tract infections (UTI), including cystitis, Pyelonephritis, Diarrhea, Meningitis in infants, Hemorrhagic colitis, Hemolytic-uremic syndrome | Human | [1,4,5,7-11,13,14] |
|  | *Flavobacterium sp.* | Negative | Through the water column and directly by fish-to-fish contact. | (Fish pathogen) Rainbow trout fry syndrome, bacterial cold-water disease | Fish | [1] |
|  | *Klebsiella pneumonia* | Negative | Direct contact with discharges from the nose and throat of infected people or by contact with infected wounds or sores on the skin | Nosocomial infections, urinary tract infections, pneumonia, septicemias, and soft tissue infections | Human | [2,6,8,9,11,14] |
|  | *Klebsiella sp.* | Negative | Through person-to-person contact (for example, from patient to patient via the contaminated hands of healthcare personnel or other persons) or, less commonly, by contamination of the environment. | Nosocomial infections | Human | [1] |
|  | *Legionella pneumophila* | Negative | Mode of infection of Legionnaires is inhaling the bacteria from water or soil, Aspiration, Soil | Legionnaire's Disease, Pontiac fever | Human | [12] |
|  | *Micrococcus luteus* | Positive | Breastfeeding mothers, direct contact with an infected person, by using a contaminated object, or by inhaling infected droplets dispersed by sneezing or coughing. | Skin infection (pruritic eruptions), recurrent bacteremia, septic shock, septic arthritis, endocarditis, meningitis, intracranial suppuration, and cavitating pneumonia in immunosuppressed patients | Human | [2,3,15,16] |
|  | *Neisseria meningitis* | Negative | Direct or indirect contact with contaminated persons or objects | Meningococcal disease including meningitis, Waterhouse-Friderichsen syndrome | Human | [3] |
|  | *Proteus mirabilis* | Negative |  | Urinary tract infections and formation of stones (48% of *P. mirabilis* strains are resistant to amoxicillin, penicillin, fluoroquinolones and other broad-range activity antibiotics.) |  | [2,3,15,16] |
|  | *Proteus vulgaris* | Negative | Genetic transformation process | UTIs, Pneumonia or Septicemia | Human | [2,3,5,7,10,11,17] |
|  | *Pseudomonas aeruginosa* | Negative | The most common disease UTI is transmitted by the migration of bacteria up the catheter along the mucosal sheath or migration up the catheter lumen from infected urine. | Pneumonia, urinary tract infections, Gastrointestinal infections, septic shock, and soft tissue or CNS infections | Human | [1-3,6,8,9,12,14-16] |
|  | *Salmonella sp.* | Negative | The most common disease UTI is transmitted by the migration of bacteria up the catheter along the mucosal sheath or migration up the catheter lumen from infected urine. | Gastroenteritis and enteric (typhoid) fever | Human | [1,13] |
|  | *Salmonella typhi* | Negative | Through contaminated hands, equipment or surfaces. | Typhoid fever-type salmonellosis (dysentery, colitis) | Human | [4] |
|  | *Salmonella typhimurium* | Negative | Contaminated food, water | Salmonellosis with gastroenteritis and enterocolitis | Human | [4,8,12] |
|  | *Shigella boydii* | Negative | Through contaminated food and water or the close contact the infected person | Diarrhoea and Shigellosis | Human | [4] |
|  | *Shigella sp.* | Negative | Through contaminated water, food, and the direct transmission of people | Shigellosis | Human | [13] |
|  | *Shigella sonnei* | Negative | From the contaminated water, food, and direct contact with infected people | Bacillary dysentery/Shigellosis | Human | [4,12] |
|  | *Staphylococcus aureus* | Positive | By eating contaminated food, drinking water and direct contact with shigella infected person | Coagulase-positive staphylococcal infections:  Localized skin infections  Diffuse skin infection (Impetigo)  Deep, localized infections  Acute infective endocarditis  Septicemia  Necrotizing pneumonia, invasive diseases  Toxinoses- Toxic shock syndrome and food poisoning | Human | [2-4,6-10,12-16] |
|  | *Staphylococcus epidermis* | Positive | From an infected wound, skin to skin contact with an infected person, contact with infected objects. | Nosocomial infection, Infections of implanted prostheses, e.g. heart valves and catheters | Human | [2,3,8,9] |
|  | *Staphylococcus saprophyticus* | Positive | Direct or indirect contact with a person who has a discharging wound or clinical infection of the respiratory or urinary tract, or who is colonised with the organism | Uncomplicated Urinary Tract Infections, Cystitis in women leading to pyelonephritis, septicemia, nephrolithiasis, and endocarditis | Human | [2,3] |
|  | *Streptococcus pneumoniae* | Positive | Inhalation of respiratory droplets, skin contact, contact with objects, surface, or dust that is contaminated with bacteria or, less commonly, transmission through food | Acute bacterial pneumonia & meningitis in adults,  Otitis media and sinusitis in children | Human | [12] |
|  | *Streptococcus pyogenes* | Positive | Bacteria infected food when going to the stomach | Streptococcal, pharyngitis, Scarlet fever, Rheumatic fever, Impetigo and erysipelas, Puerperal fever, pyoderma to severe and life-threatening invasive infections, Necrotizing fasciitis, dizziness, and red rash at the wound site | Human | [2,3,12,13,15,16] |
|  | *Vibrio alginolyticus* | Negative |  | Wound infections, Ear infections, chronic  diarrhoea in a patient with AIDS, conjunctivitis and post-traumatic intracranial  infection | Human, animal, fish | [1,4] |
|  | *Vibrio cholerae* | Negative | Ingestion of bacterial contaminated food and water, through faecal. | Cholera, profuse watery diarrhoea, vomiting, and muscle cramps | Human | [1,4] |
|  | *Vibrio parahaemolyticus* | Negative | Eating raw or undercooked shellfish, mainly oysters get infected in the stomach. | Seafood-associated gastroenteritis | Human | [1] |
|  | **Virus** |  |  |  |  |  |
|  | Dengue Virus Serotype 1 (DENV-1) |  | Through the mosquito bites | Dengue fever | Human | [18] |
|  | Herpes Simplex Virus type 1 (HSV-1) |  | The virus is spread by contact, the usual site for the implantation is skin or mucous membrane. | Mucocutaneous infection (most common), including genital herpes  Ocular infection (including herpes keratitis)  Central nervous system (CNS) infection  Neonatal herpes | Human | [19] |
|  | Human Immune Deficiency Virus type 1 (HIV-1) |  | HIV is transmitted through close contact with a body fluid that contains the virus or cells infected with the virus (such as blood, semen, or vaginal fluids). | Destroy the immune system | Human | [20] |
|  | Influenza A |  | Transmit from birds to human | Respiratory disease | Human and animal | [21] |
|  | **Fungi** |  |  |  |  |  |
|  | *Aspergillus niger* |  | By infecting onion seedlings, Damaged epithelial lining and respiratory tract system in human | A black mould of onions, pulmonary aspergillosis in human, otomycosis | Human, Animal and Plants | [22-24] |
|  | *Aspergillus oryzae* |  |  |  |  | [24] |
|  | *Candida albicans* |  | Alterations in the host microbiota, changes in the host immune response, or variations in the local environment | Superficial mucosal and dermal infections, such as thrush, vaginal yeast infections | Human | [23] |
|  | *Candida krusei* |  |  |  |  | [23] |
|  | *Candida tropicalis* |  | Through the bloodstream | It is particularly virulent in neutropenic hosts commonly with hematogenous seeding to peripheral organs. | Human and domestic animal | [23] |
|  | *Helminthosporium oryzae* |  | During warm and moist periods. reddish-brown to purplish-black spots on leaves is found | Black helminthosporium, | Plant | [25] |
|  | *Helminthosporium sativum* |  | Periods of temporary drought stress attacks the leaf and root | Leaf spot and root rot of Kentucky bluegrass | Plant |  |
|  | *Microsporum canis* |  | Ringworm is fungal infection through the skin, tinea capitis infected through the scalp, tinea corporis fungal infection through the arms and legs | Ringworm, tinea capitis, tinea corporis | Animal and human | [23] |
|  | *Penicillium chrysogenum* |  | Pulmonary inflammation lying on a spectrum between lung abscess and pulmonary gangrene | Necrotizing pneumonia a caused by *P. chrysoge* num in a patient with cancer. | Human | [24] |
|  | *Penicillium sp.* |  | A fungal infection that affects one, or occasionally both, of the ears | Chronic granulomatous disorder (CGD ), Superficial infection (keratitis and otomycosis) | human | [24] |
|  | *Trichophyton mentagrophytes* |  | Spread by the spore | Skin infection is known as Dermatophytosis or ringworm. | Animal, human. But It can be transferred from animal to human. | [23] |
|  | *Trichophyton rubrum* |  | Direct contact, by contact with infected particles, shed by the host, and by contact with the fungi's spores | Tinea, athlete’s foot, ringworm, joke itch, scalp | Human | [23] |
|  | **Parasites** |  |  |  |  |  |
|  | *Aedes aegypti* |  | Spread to people through the bites of infected Aedes | Zika, dengue, chikungunya, and yellow fever | Human | [26,27] |
|  | *Anopheles subpictus* |  | Spread to people through the bites of mosquito | malaria | Human, animals (zoophily) | [28] |
|  | *Ascaris lumbricoides* |  | Eating food or drink contaminated with *Ascaris* eggs from faeces | Ascariasis | Human | [29] |
|  | *Cluex quinquefasciatus* |  | Causes infection through biting during a blood meal. | Avian malaria, avian pox virus, human filariasis, encephalitis, | Animal, birds, human | [26,27] |
|  | *Cluex tritaeniorhynchus* |  | Through the mosquito bites | Japanese Encephalitis Virus JEV | Domestic swine and ardeid birds and human | [28] |
|  | *Leishmania infantum* |  | The primary way is through the bite of infected female phlebotomine sandflies. | leishmaniasis | Human | [30] |
|  | *Plasmodium falciparum* |  | Mosquito carrier | Malaria | Human | [31] |
|  | *Plasmodium berghei* |  | Mosquito carrier | Malaria | Human | [31] |
|  | *Trypanosoma brucei brucei* |  | Transmitted by the tsetse fly (*Glossina* species) .to be ensured by the parasite's presence in any body fluid. | African Trypanosomiasis, also known as “sleeping sickness.” | Human | [30] |
|  | *Trypanosoma cruzi* |  | Insect vectors | Chagas disease | Animal and peoples | [30] |

**Reference**

1. Wei, L.S.; Wee, W.; Siong, J.Y.F.; Syamsumir, D.F. Characterizaion of antimicrobial, antioxidant, anticancer properties and chemical composition of Malaysian *Andrographis paniculata* leaf extract. *Pharmacologyonline* **2011**, *2*, 996-1002.

2. Sule, A.; Ahmed, Q.U.; Samah, O.A.; Omar, M.N. Screening for Antibacterial Activity of Andrographis paniculata Used in Malaysian Folkloric Medicine: A Possible Alternative for the Treatment of Skin Infections. *Ethnobotanical Leaflets* **2010**, *14*, 445-456.

3. Sule, A.; Ahmed, Q.U.; Samah, O.A.; Omar, M.N. Bacteriostatic and bactericidal activities of Andrographis paniculata extracts on skin disease causing pathogenic bacteria. *Journal of Medicinal Plants Research* **2011**, *5*, 7-14.

4. Mishra, U.S.; Mishra, A.; Kumari, R.; Murthy, P.N.; Naik, B.S. Antibacterial Activity of Ethanol Extract of Andrographis paniculata. *Indian J Pharm Sci* **2009**, *71*, 436-438, doi:10.4103/0250-474X.57294.

5. Radhika, P.; Sastry, B.S.; Madhu, H.B. Antimicrobial screening of Andrographis paniculata (Acanthaceae) root extracts. *Research Journal of Biotechnology* **2008**, *3*, 62-63.

6. Kataky, A.; Handique, P.J. Micropropagation and screening of antioxidant potential of Andrographis paniculata (Burm. f) Nees. *Journal of Hill Agriculture* **2010**, *1*, 13-18.

7. Nakanishi, K.; Sasaki, S.; Kiang, A.K.; Goh, J.; Kakisawa, H.; Ohashi, M.; Goto, M.; Watanabe, J.; Yokotani, H.; Matsumura, C., et al. Phytochemical survey of Malaysian plants preliminary chemical and pharmacological screening. *Chem Pharm Bull (Tokyo)* **1965**, *13*, 882-890, doi:10.1248/cpb.13.882.

8. Roy, S.; Rao, K.; Bhuvaneswari, C.; Giri, A.; Mangamoori, L.N. Phytochemical analysis of Andrographis paniculata extract and its antimicrobial activity. *World journal of microbiology & biotechnology* **2010**, *26*, 85-91, doi:10.1007/s11274-009-0146-8.

9. Sahalan, A.Z.; Sulaiman, N.; Mohammed, N.; Ambia, K.M.; Lian, H.H. Antibacterial activity of Andrographis paniculata and Euphorbia hirta methanol extracts. *Jurnal Sains Kesihatan Malaysia* **2007**, *5*, 1-8.

10. Radhika, P.; Lakshmi, K.R. Antimicrobial Activity of the Chloroform Extracts of the Root and the Stem of Andrographis paniculata Nees. *International Research Journal of Microbiology* **2010**, *1*, 37-39.

11. Aniel Kumar, O.; Mutyala Naidu, L.; Raja Rao, K. In vitro antibacterial activity in the extracts of Andrographis paniculata Burm. F. *International Journal of PharmTech Research* **2010**, *2*, 1383-1385.

12. Xu, Y.; Marshall, R.L.; Mukkur, T.K. An Investigation on the Antimicrobial Activity of Andrographis paniculata Extracts and Andrographolide in vitro. *Asian Journal of Plant Sciences* **2006**, *5*, 527-530.

13. Leelarasamee, A.; Trakulsomboon, S.; Sittisomwong, N. Undetectable anti-bacterial activity of Andrographis paniculata (Burma) wall. ex ness. *J Med Assoc Thai* **1990**, *73*, 299-304.

14. Zaidan, M.; Noor Rain, A.; Badrul, A.; Adlin, A.; Norazah, A.; Zakiah, I. In vitro screening of five local medicinal plants for antibacterial activity using disc diffusion method. *Trop Biomed* **2005**, *22*, 165-170.

15. Ahmed, Q.U.; Samah, O.A.; Sule, A. Andrographis paniculata (Burm.f) Wall. ex Ness: A Potent Antibacterial Plant. In *Antimicrobial Agents*, Bobbarala, V., Ed. InTech: 2012; 10.5772/34420pp 345-360.

16. Sule, A.; Ahmed, Q.U.; Samah, O.A.; Omar, M.N.; Hassan, N.M.; Kamal, L.Z.M.; Yarmo, M.A. Bioassay guided isolation of antibacterial compounds from *Andrographis paniculata* (Burm.f.) Wall. ex Nees (Hempedeu bumi). *American J Applied Sci.* **2011**, *8*, 525-534.

17. Chuthaputti, A.; Pornpatkul, V.; Suwankiri, U. The Efficacy of Andrographis paniculata (Burm. f.) Wall. ex Nees for the Relief of the Symptoms of Influenza. *Journal of Thai Traditional & Alternative Medicine* **2007**, *5*, 1-10.

18. Tang, L.I.; Ling, A.P.; Koh, R.Y.; Chye, S.M.; Voon, K.G. Screening of anti-dengue activity in methanolic extracts of medicinal plants. *BMC Complement Altern Med* **2012**, *12*, 3, doi:10.1186/1472-6882-12-3.

19. Aromdee, C.; Suebsasana, S.; Ekalaksananan, T.; Pientong, C.; Thongchai, S. Stage of Action of Naturally Occurring Andrographolides and Their Semisynthetic Analogues against Herpes Simplex Virus Type 1 in Vitro. *Planta Medica* **2011**, *77*, 915-921, doi:10.1055/s-0030-1250659.

20. Xu, H.X.; Wan, M.; Loh, B.N.; Kon, O.L.; Chow, P.W.; Sim, K.Y. Screening of traditional medicines for their inhibitory activity against HIV-1 protease. *Phytotherapy Research* **1996**, *10*, 207-210.

21. Chen, J.X.; Xue, H.J.; Ye, W.C.; Fang, B.H.; Liu, Y.H.; Yuan, S.H.; Yu, P.; Wang, Y.Q. Activity of andrographolide and its derivatives against influenza virus in vivo and in vitro. *Biol Pharm Bull* **2009**, *32*, 1385-1391, doi:10.1248/bpb.32.1385.

22. Bobbarala, V.; Katikala, P.K.; Naidu, K.C.; Penumajji, S. Antifungal activity of selected plant extracts against phytopathogenic fungi Aspergillus niger F2723. *Indian Journal of Science and Technology* **2009**, *2*, 87-90.

23. Sule, A.; Ahmed, Q.U.; Latip, J.; Samah, O.A.; Omar, M.N.; Umar, A.; Dogarai, B.B. Antifungal activity of Andrographis paniculata extracts and active principles against skin pathogenic fungal strains in vitro. *Pharm Biol* **2012**, *50*, 850-856, doi:10.3109/13880209.2011.641021.

24. Wanchaitanawong, P.; Chaungwanit, P.; Poovarodom, N.; Nitisinprasert, S. In vitro antifungal activity of Thai herb and spice extracts against food spoilage fungi. *Kasetsart Journal (Natural Science)* **2005**, *39*, 400-405.

25. Alagesaboopathi, C.; Balu, S. Antifungal activity of some species of Andrographis wallich ex Nees on Helminthosporium oryzae Breda dehaan. *Journal of Economic and Taxonomic Botany* **2000**, *24*, 705-707.

26. Govindarajan, M. Evaluation of Andrographis paniculata Burm.f. (Family:Acanthaceae) extracts against Culex quinquefasciatus (Say.) and Aedes aegypti (Linn.) (Diptera:Culicidae). *Asian Pac J Trop Med* **2011**, *4*, 176-181, doi:10.1016/S1995-7645(11)60064-3.

27. Govindarajan, M.; Sivakumar, R. Adulticidal and repellent properties of indigenous plant extracts against Culex quinquefasciatus and Aedes aegypti (Diptera: Culicidae). *Parasitol Res* **2012**, *110*, 1607-1620, doi:10.1007/s00436-011-2669-9.

28. Elango, G.; Rahuman, A.A.; Bagavan, A.; Kamaraj, C.; Zahir, A.A.; Venkatesan, C. Laboratory study on larvicidal activity of indigenous plant extracts against Anopheles subpictus and Culex tritaeniorhynchus. *Parasitol Res* **2009**, *104*, 1381-1388, doi:10.1007/s00436-009-1339-7.

29. Raj, R.K. Screening of some indigenous plants for anthelmintic action against human Ascaris lumbricoides. *Indian journal of physiology and pharmacology* **1974**, *18*, 129-131.

30. Dua, V.K.; Verma, G.; Dash, A.P. In Vitro Antiprotozoal Activity of Some Xanthones Isolated from the Roots of Andrographis paniculata. *Phytotherapy Research* **2009**, *23*, 126-128, doi:10.1002/ptr.2556.

31. Dua, V.K.; Qjha, V.P.; Roy, R.; Joshi, B.C.; Valecha, N.; Devi, C.U.; Bhatnagar, M.C.; Sharma, V.P.; Subbatao, S.K. Anti-malarial activity of some xanthones isolated from the roots of *Andrographis paniculata*. *Journal of Ethnopharmacology* **2004**, *95*, 247-251, doi:10.1016/j.jep.2004.07.008.
