# Supplementary material for: Andrographis paniculata (Burm. f.) Wall. ex Nees: An Updated Review of Phytochemistry, Antimicrobial Pharmacology, and Clinical Safety and Efficacy
Source: Life (Basel). 2021 Apr 16;11(4):348. doi: 10.3390/life11040348 (PMC8072717; doi:10.3390/life11040348)
Supplement: Supplementary file 1 [file life-11-00348-s001.zip › life-1140970/Supplementary Table S2.docx]

Table S2. Isolated pure metabolites of *A. paniculata* and the part used.

| **No.** | **Name** | **Part Used** | **Reference** |
| --- | --- | --- | --- |
| ***ent*-Labdane Diterpenoids** | | | |
|  | (13*R*, 14*R*) 3,13,14,19-tetrahydroxy-ent-labda-8 (17), 11-dien-16,15-olide | L | [1] |
|  | 12-*epi*-14-deoxy-12-methoxy-andrographolide | AeP | [2] |
|  | 12*R*,13*R*-hydroxyandrographolide | AeP | [3] |
|  | 12*S*,13*S*-hydroxyandrographolide | AeP | [3] |
|  | 12*S*-hydroxyandrographolide | AeP | [4] |
|  | 13,14,15,16-tetranor-ent-labd-8(17)-ene-3,12,19-triol | AeP | [3,5] |
|  | 14-deoxy-11,12-didehydroandrographiside | AeP | [2,4,6,7] |
|  | 14-deoxy-11,12-didehydroandrographolide | AeP, L | [1-23]] |
|  | 14-deoxy-11-hydroandrographolide | AeP | [2,6,10,23-25] |
|  | 14-deoxy-11-oxoandrographolide | AeP | [6] |
|  | 14-deoxy-12-hydroandrographolide | AeP | [2,4,16,24]] |
|  | 14-deoxy-12-methoxyandrographolide | AeP, L | [2,9,17] |
|  | 14-deoxy-14,15-didehydroandrographolide | AeP, WP | [8,15] |
|  | 14-deoxy-15-isopropylidene-11,12, didehydrographolide | AeP, R | [20-21] |
|  | 14-deoxy-15-methoxy-andrographolide | AeP | [46] |
|  | 14-deoxy-17-hydroxyandro-grapholide | AeP | [23, [24]] |
|  | 14-deoxyandrographolide | AeP, L | [1-6,9,10,12,14,16-18,20,21]] |
|  | 14-deoxyandrographoside | L | [1,6] |
|  | 14-deoxy-11(R)-hydroxyandrographiside | AeP | [6] |
|  | 14-*epi*-andrographolide | AeP | [2] |
|  | 19-[(*β*-*D*-glucopyranosyl)oxy]-19-oxo-ent-labda-8(17),13-dien-16,15-olide | AeP | [24] |
|  | 19-hydroxy-3-oxo-ent-labda-8(17),11,13-trien-16,15-olide | AeP | [24] |
|  | 19-*O*-[ *β* -D-apiofuranosyl(1→2)-*β*-*D*-glucopyranoyl]-3,14-dideoxyandrographolide | AeP | [23] |
|  | 19-*O*-acetyl-14-deoxy-11,12 –didehydro-andrographolide | WP | [8] |
|  | 19-*O*-*β*-*D*-glucopyranosyl-ent-labda-8(17),13-dien-15,16,19-triol | AeP | [6,23] |
|  | 21-nor-3,19–isopropylidine-14-deoxy-ent-labda-8(17),13-dien-16,15-olide | L | [38] |
|  | 3,14-dideoxyandrographolide | AeP | [3] |
|  | 3,15,19-trihydroxy-ent-labda-8(17),13-dien-16-oic acid | AeP | [3,5] |
|  | 3,18,19-trihydroxy-entlabda-8(17),13-dien-16,15-olide | AeP | [24] |
|  | 3,19-dihydroxy-14,15,16-trinor-ent-labda-8(17),11-dien-13-oic acid | AeP | [3,5] |
|  | 3,19-dihydroxy-15-epi-methoxy-8(17),11,13-ent-labdatrien-16,15-olide | NS | [24] |
|  | 3,19-dihydroxy-15-epi-methoxy-8(17),12-ent-labdadien-16,15-olide | NS | [24] |
|  | 3,19-dihydroxy-15-methoxy-ent-labda-8(17),11,13-trien-16,15-olide | AeP | [3,5,24] |
|  | 3,19-dihydroxy-ent-labda-8(17),12-dien-16,15-olide | AeP | [5,6,24]] |
|  | 3-dehydroandrographolide | NS | [24] |
|  | 3,14-deoxy-17β-hydroxy  andrographolide | AeP | [6] |
|  | 3-deoxyandrographoside | AeP | [46] |
|  | 3-oxo-14-Deoxy-11,12, didehydroandrographolide | AeP | [6] |
|  | 3-oxo-14-deoxyandrographolide | AeP | [3] |
|  | 3-oxo-14-deoxyandrographolide | AeP | [3] |
|  | 3-oxo-*ent*-cleroda-8(17),11,13-trien-16,15-olide | AeP | [44] |
|  | 3-*O*-*β*-D-glucopyranosyl-14,19-dideoxyandrographolide | AeP | [23] |
|  | 3-*O*-*β*-*D*-glucopyranosyl-andrographolide | AeP | [4,6] |
|  | 3-*O*-*β*-*D*-glucosyl-14-deoxy-11,12-didehydroandrographiside | AeP | [24,26] |
|  | 3-*O*-*β*-*D*-glucosyl-14-deoxyandrographiside | AeP | [24,26] |
|  | 6′-acetylneoandrographolide | AeP | [2,6] |
|  | 6-epi-8-O-Acetylharpagide | L | [37] |
|  | 7*R*-hydroxy-14-deoxyandrographolide | AeP | [3] |
|  | 7*S*-hydroxy-14-deoxyandrographolide | AeP | [3] |
|  | 8,17-epoxy-14-deoxyandrographolide | AeP | [4,6] |
|  | Andrograpanin | AeP, L | [1,4,9,12,18,27,28] |
|  | Andrograpenoid A | AeP | [6] |
|  | Andrograpenoid B | AeP | [6] |
|  | Andrographatoside | AeP | [23, [6] |
|  | Andrographic acid | NS | [13] |
|  | Andrographic acid methyl ester | AeP | [13] |
|  | Andrographiside | AeP, L, WP | [1-7,10,23,25] |
|  | Andrographolide | AeP, L, R, WP | [1-7,9-18,20,21,23-25,27,29] |
|  | Andropanolide | L | [25, 43] |
|  | Andropanolide B | AeP | [43] |
|  | Andropanoside | L | [16, 37] |
|  | Bis-andrographolide A | AeP | [2] |
|  | Bis-andrographolide B | AeP | [2] |
|  | Bis-andrographolide C | AeP | [2] |
|  | Bis-andrographolide D | AeP | [2] |
|  | Bis-andrographolide ether | AeP | [22] |
|  | Curvifloruside F | R | [37, 39] |
|  | Deoxyandrographolide-19-*β*-*D*-glucoside | L, AeP | [2,5,23-25,30] |
|  | Echiodinin | Callus | [31] |
|  | ent-labda-8(17),13-diene-15,16,19-triol | AeP | [3,5] |
|  | Isoandrographiside | AeP | [6] |
|  | Isoandrographolide | AeP, L, R, WP | [2,3,6,11,12,15,17,18,32,33] |
|  | Loliolide | AeP | [18] |
|  | Neoandrographolide | AeP, L, R, WP | [1-3,6,7,9,12,14-17,20,21,23-25,27,32,33] |
|  | Oleanolic acid | AeP | [6] |
|  | Pashanone glucoside | AeP | [13] |
|  | Procumbide | L | [7] |
|  | Procumboside | L | [7] |
|  | Wightiolide | AeP | [6] |
| **Flavonoids** | | | |
|  | (-)-Onysilin | WP | [17] |
|  | (2*R*)-7,8-dimethoxyflavanone-5-*O*-*β*-*D*-glucopyranoside | NS | [13] |
|  | 5,2′-dihydroxy-7,8-dimethoxy-flavanone (dihydroskullcapflavone I) | WP, AeP | [10,17,18] |
|  | 5,2′-dihydroxy-7,8-dimethoxyflavone (2) | Callus | [34] |
|  | 5,3′,4′-Trihydroxy-7-methoxy-flavone | AeP | [18,35] |
|  | 5,4′-dihydroxy-7,8,2′,3′-tetramethoxy-flavone | AeP | [36] |
|  | 5,4′-dihydroxy-7-methoxy-8-*O*-*β*-*D*-glucopyranosyloxyflavone | AeP | [36] |
|  | 5,4′-Dihydroxy-7-*O*-*β*-*D*-glucopyranosyl-oxyflavone | AeP | [36] |
|  | 5,4′-dihydroxy-7-*O*-*β*-*D*-pyranglycuronate butyl ester | AeP | [36] |
|  | 5,7,2′,3′-tetramethoxyflavanone | WP | [10] |
|  | 5,7,8-trimethoxydihydroflavone | AeP | [36] |
|  | 5,7,8-trimethoxyflavanone | AeP | [18] |
|  | 5-hydroxy-3,7,8,2′-tetramethoxyflavone | R | [37] |
|  | 5-hydroxy-7,2′,3′-trimethoxyflavone | WP | [10] |
|  | 5-hydroxy-7,2′,6′-trimethoxy-flavone | AeP, WP, R | [10,11] |
|  | 5-hydroxy-7,8,2′,5′-tetramethoxy-flavone | AeP, WP | [10,17,36] |
|  | 5-hydroxy-7,8,2′3′-tetramethoxyflavone | AeP, R, WP | [10,35,36] |
|  | 5-hydroxy-7,8,2′-trimethoxy-flavone (skullcapflavone I 2′-methyl ether) | AeP, Callus, R, WP | [10,11,34,38] |
|  | 5-hydroxy-7,8-dimethoxyflavanone  (7-O-methyldihydrowogonin) | R, AeP & WP | [10,36,37] |
|  | 5-hydroxy-7,8-dimethoxyflavone  (7-*O*-methylwogonin) | AeP, Callus, R, WP | [10-12,17,34,35,37,38] |
|  | 7,8,2′,5′-tetramethoxy-flavone-5-*O*-β -D-glucopyranoside | NS [13], AeP | [13,36] |
|  | Andrographidine A | AeP, R, L | [7,35,36] |
|  | Andrographidine B | R | [35] |
|  | Andrographidine C  (7-*O*-methylwogonin-5-glucoside) | AeP, R, WP | [10,11,35,36] |
|  | Andrographidine D | R | [35] |
|  | Andrographidine E | R | [35] |
|  | Andrographidine F | R | [35] |
|  | Andropaniculoside A | AeP, WP, L | [7,17,29,38] |
|  | Andropaniculosin A | WP | [17] |
|  | Apigenin | AeP, WP | [17,38,39] |
|  | Apigenin-7-*O*-*β*-*D*-methylglucuronide | WP | [17] |
|  | Cosmosiin | WP | [17] |
|  | Ermanin | AeP | [18] |
|  | Isoswertisin | WP | [17] |
|  | Kaempferol (tetrahydroxyflavon) | L | [40] |
|  | Luteolin | AeP | [38] |
|  | Pinocembrin | AeP | [18] |
|  | Pinostrobin | AeP | [18] |
|  | Quercetin | WP | [17] |
|  | Scutellarin-6-*O*-*β*-*D*-glucoside-7-methyl ether | AeP, WP | [17] |
|  | Skullcapflavone I 2′-*O*-glucoside | R, WP | [10,11] |
| **Xanthones** | | | |
|  | 1,2-dihydroxy-6,8-dimethoxyxanthone | R | [41,42] |
|  | 1,8-dihydroxy-3,7-dimethoxyxanthone | R | [41,42] |
|  | 3,7,8-Trimethoxy-1-hydroxyxanthone | R | [41,42] |
|  | 4,8-dihydroxy-2,7-dimethoxyxanthone | R | [41,42] |
| **Rare Noriridoids** | | | |
|  | Andrographidoid A | R | [43] |
|  | Andrographidoid B | R | [43] |
|  | Andrographidoid C | R | [43] |
|  | Andrographidoid D | R | [43] |
|  | Andrographidoid E | R | [43] |
| **Steroids** | | | |
|  | Ergosterol peroxide | AeP | [18] |
|  | Stigmasterol | WP | [8] |
|  | Sitosterol | AeP, L, WP | [8,10,18] |
| **Quinic Acid Derivatives** | | | |
|  | 3,4-dicaffeoylquinic acid | WP | [17] |
|  | Adipic acid | WP | [17] |
|  | Caffeic acid | WP | [10] |
|  | Chlorogenic acid | WP | [10] |
|  | Cinnamic acid | WP | [17] |
|  | Ferulic acid | L, WP | [10,16] |
|  | Methyl-3,4-dicaffeoylquinate | WP | [10,16,17] |
|  | Stearic acid | AeP | [18] |
| **Other Compounds** | | | |
|  | Acanthoside B | L | [7] |
|  | Bis(2-ethylhexyl) phthalate | AeP | [18] |
|  | Roseooside | L | [29] |

WP: Whole plant, AeP: Aerial part, L: Leaves, R: Root, NS: Not specify plant part

**Reference**

1. Xu, C.; Chou, G.X.; Wang, Z.T. A new diterpene from the leaves of Andrographis paniculata Nees. *Fitoterapia* **2010**, *81*, 610-613, doi:10.1016/j.fitote.2010.03.003.

2. Matsuda, T.; Kuroyanagi, M.; Sugiyama, S.; Umehara, K.; Ueno, A.; Nishi, K. Cell differentiation-inducing diterpenes from Andrographis paniculata Nees. *Chem Pharm Bull (Tokyo)* **1994**, *42*, 1216-1225, doi:10.1248/cpb.42.1216.

3. Chen, L.; Zhu, H.; Wang, R.; Zhou, K.; Jing, Y.; Qiu, F. ent-Labdane diterpenoid lactone stereoisomers from Andrographis paniculata. *J Nat Prod* **2008**, *71*, 852-855, doi:10.1021/np0704452.

4. Shen, Y.H.; Li, R.T.; Xiao, W.L.; Xu, G.; Lin, Z.W.; Zhao, Q.S.; Sun, H.D. ent-Labdane diterpenoids from Andrographis paniculata. *J Nat Prod* **2006**, *69*, 319-322, doi:10.1021/np050160u.

5. Chen, L.X.; Qiu, F.; Wei, H.; Qu, G.X.; Yao, X.S. Nine new ent-labdane diterpenoids from the aerial parts of Andrographis paniculata. *Helvetica Chimica Acta* **2006**, *89*, 2654-2664, doi:DOI 10.1002/hlca.200690237.

6. Wen, Q.; Jin, X.; Lu, Y.; Chen, D.F. Anticomplement ent-labdane diterpenoids from the aerial parts of Andrographis paniculata. *Fitoterapia* **2020**, *142*, 104528, doi:10.1016/j.fitote.2020.104528.

7. Hapuarachchi, S.D.; Ali, Z.; Abe, N.; Sugandhika, S.T.; Sandun, S.T.; Khan, I.A. Andrographidine G, a new flavone glucoside from Andrographis paniculata. *Nat Prod Commun* **2013**, *8*, 333-334, doi:10.1177/1934578x1300800314.

8. Chao, W.W.; Kuo, Y.H.; Lin, B.F. Anti-inflammatory activity of new compounds from Andrographis paniculata by NF-kappaB transactivation inhibition. *J Agric Food Chem* **2010**, *58*, 2505-2512, doi:10.1021/jf903629j.

9. Fujita, T.; Fujitani, R.; Takeda, Y.; Takaishi, Y.; Yamada, T.; Kido, M.; Miura, I. On the Diterpenoids of Andrographis-Paniculata - X-Ray Crystallographic Analysis of Andrographolide and Structure Determination of New Minor Diterpenoids. *Chemical & Pharmaceutical Bulletin* **1984**, *32*, 2117-2125, doi:10.1248/cpb.32.2117.

10. Rao, Y.K.; Vimalamma, G.; Rao, C.V.; Tzeng, Y.M. Flavonoids and andrographolides from Andrographis paniculata. *Phytochemistry* **2004**, *65*, 2317-2321, doi:10.1016/j.phytochem.2004.05.008.

11. Reddy, M.K.; Reddy, M.V.; Gunasekar, D.; Murthy, M.M.; Caux, C.; Bodo, B. A flavone and an unusual 23-carbon terpenoid from Andrographis paniculata. *Phytochemistry* **2003**, *62*, 1271-1275, doi:10.1016/s0031-9422(03)00051-7.

12. Reddy, V.L.; Reddy, S.M.; Ravikanth, V.; Krishnaiah, P.; Goud, T.V.; Rao, T.P.; Ram, T.S.; Gonnade, R.G.; Bhadbhade, M.; Venkateswarlu, Y. A new bis-andrographolide ether from Andrographis paniculata nees and evaluation of anti-HIV activity. *Nat Prod Res* **2005**, *19*, 223-230, doi:10.1080/14786410410001709197.

13. Hanh, T.T.H.; My, N.T.T.; Cham, P.T.; Quang, T.H.; Cuong, N.X.; Huong, T.T.; Nam, N.H.; Minh, C.V. Diterpenoids and Flavonoids from Andrographis paniculata. *Chem Pharm Bull (Tokyo)* **2020**, *68*, 96-99, doi:10.1248/cpb.c19-00662.

14. Awang, K.; Abdullah, N.H.; Hadi, A.H.; Fong, Y.S. Cardiovascular activity of labdane diterpenes from Andrographis paniculata in isolated rat hearts. *J Biomed Biotechnol* **2012**, *2012*, 876458, doi:10.1155/2012/876458.

15. Geethangili, M.; Rao, Y.K.; Fang, S.H.; Tzeng, Y.M. Cytotoxic constituents from Andrographis paniculata induce cell cycle arrest in jurkat cells. *Phytother Res* **2008**, *22*, 1336-1341, doi:10.1002/ptr.2493.

16. Radhika, P.; Prasad, Y.R.; Sowjanya, K. A new diterpene from the leaves of Andrographis paniculata Nees. *Nat Prod Commun* **2012**, *7*, 485-486, doi:10.1177/1934578x1200700417.

17. Wu, T.S.; Chern, H.J.; Damu, A.G.; Kuo, P.C.; Su, C.R.; Lee, E.J.; Teng, C.M. Flavonoids and ent-labdane diterpenoids from Andrographis paniculata and their antiplatelet aggregatory and vasorelaxing effects. *J Asian Nat Prod Res* **2008**, *10*, 17-24, doi:10.1080/10286020701273627.

18. Lee, S.; Morita, H.; Tezuka, Y. Preferentially Cytotoxic Constituents of Andrographis paniculata and their Preferential Cytotoxicity against Human Pancreatic Cancer Cell Lines. *Natural Product Communications* **2015**, *10*, 1153-1158, doi:10.1177/1934578X1501000704.

19. Paczosa, M.K.; Mecsas, J. Klebsiella pneumoniae: Going on the Offense with a Strong Defense. *Microbiol Mol Biol Rev* **2016**, *80*, 629-661, doi:10.1128/MMBR.00078-15.

20. Pholphana, N.; Rangkadilok, N.; Saehun, J.; Ritruechai, S.; Satayavivad, J. Changes in the contents of four active diterpenoids at different growth stages in Andrographis paniculata (Burm.f.) Nees (Chuanxinlian). *Chin Med* **2013**, *8*, 2, doi:10.1186/1749-8546-8-2.

21. Suriyo, T.; Pholphana, N.; Rangkadilok, N.; Thiantanawat, A.; Watcharasit, P.; Satayavivad, J. Andrographis paniculata extracts and major constituent diterpenoids inhibit growth of intrahepatic cholangiocarcinoma cells by inducing cell cycle arrest and apoptosis. *Planta Med* **2014**, *80*, 533-543, doi:10.1055/s-0034-1368399.

22. Wang, G.Y.; Wen, T.; Liu, F.F.; Tian, H.Y.; Chun-Lin, F.; Huang, X.J.; Ye, W.C.; Wang, Y. Two new diterpenoid lactones isolated from Andrographis paniculata. *Chin J Nat Med* **2017**, *15*, 458-462, doi:10.1016/S1875-5364(17)30068-7.

23. Zou, Q.Y.; Li, N.; Dan, C.; Deng, W.L.; Peng, S.L.; Ding, L.S. A new ent-labdane diterpenoid from Andrographis paniculata. *Chinese Chemical Letters* **2010**, *21*, 1091-1093, doi:10.1016/j.cclet.2010.05.002.

24. Gan, L.; Zheng, Y.; Deng, L.; Sun, P.; Ye, J.; Wei, X.; Liu, F.; Yu, L.; Ye, W.; Fan, C., et al. Diterpenoid Lactones with Anti-Inflammatory Effects from the Aerial Parts of Andrographis paniculata. *Molecules* **2019**, *24*, 2726, doi:10.3390/molecules24152726.

25. Wang, Y.Q.; Wu, Z.F.; Ke, G.; Yang, M. An effective vacuum assisted extraction method for the optimization of labdane diterpenoids from Andrographis paniculata by response surface methodology. *Molecules* **2014**, *20*, 430-445, doi:10.3390/molecules20010430.

26. Zhou, K.L.; Chen, L.X.; Zhuang, Y.L.; Wang, N.L.; Yao, X.S.; Qiu, F. Two new ent-labdane diterpenoid glycosides from the aerial parts of Andrographis paniculata. *J Asian Nat Prod Res* **2008**, *10*, 939-943, doi:10.1080/10286020802217432.

27. Pfisterer, P.H.; Rollinger, J.M.; Schyschka, L.; Rudy, A.; Vollmar, A.M.; Stuppner, H. Neoandrographolide from Andrographis paniculata as a potential natural chemosensitizer. *Planta Med* **2010**, *76*, 1698-1700, doi:10.1055/s-0030-1249876.

28. Wang, C.H.; Li, W.; Qiu, R.X.; Jiang, M.M.; Li, G.Q. A new diterpenoid from the aerial parts of Andrographis paniculata. *Nat Prod Commun* **2014**, *9*, 13-14, doi:10.1177/1934578x1400900105.

29. Zhang, L.; Liu, Q.; Yu, J.; Zeng, H.; Jiang, S.; Chen, X. Separation of five compounds from leaves of Andrographis paniculata (Burm. f.) Nees by off-line two-dimensional high-speed counter-current chromatography combined with gradient and recycling elution. *J Sep Sci* **2015**, *38*, 1476-1483, doi:10.1002/jssc.201401458.

30. Weiming, C.; Xiaotian, L. Deoxyandrographolide-19β-D-Glucoside from the Leaves of Andrographis paniculata. *Planta Med* **1982**, *45*, 245-246.

31. Arifullah, M.; Namsa, N.D.; Mandal, M.; Chiruvella, K.K.; Vikrama, P.; Gopal, G.R. Evaluation of anti-bacterial and anti-oxidant potential of andrographolide and echiodinin isolated from callus culture of Andrographis paniculata Nees. *Asian Pac J Trop Biomed* **2013**, *3*, 604-610; discussion 609-610, doi:10.1016/S2221-1691(13)60123-9.

32. Pramanick, S.; Banerjee, S.; Achari, B.; Das, B.; Sen, A.K., Sr.; Mukhopadhyay, S.; Neuman, A.; Prange, T. Andropanolide and isoandrographolide, minor diterpenoids from Andrographis paniculata: structure and X-ray crystallographic analysis. *J Nat Prod* **2006**, *69*, 403-405, doi:10.1021/np050211n.

33. Li, W.; Xu, X.; Zhang, H.; Ma, C.; Fong, H.; van Breemen, R.; Fitzloff, J. Secondary metabolites from Andrographis paniculata. *Chem Pharm Bull (Tokyo)* **2007**, *55*, 455-458, doi:10.1248/cpb.55.455.

34. Jalal, M.A.F.; Overton, K.H.; Rycroft, D.S. Formation of three new flavones by differentiating callus cultures of andrographis paniculata. *Phytochemistry* **1979**, *18*, 149-151, doi:10.1016/s0031-9422(00)90934-8.

35. Kuroyanagi, M.; Sato, M.; Ueno, A.; Nishi, K. Flavonoids from Andrographis-Paniculata. *Chemical & Pharmaceutical Bulletin* **1987**, *35*, 4429-4435, doi:10.1248/cpb.35.4429.

36. Chen, H.; Ma, Y.B.; Huang, X.Y.; Geng, C.A.; Zhao, Y.; Wang, L.J.; Guo, R.H.; Liang, W.J.; Zhang, X.M.; Chen, J.J. Synthesis, structure-activity relationships and biological evaluation of dehydroandrographolide and andrographolide derivatives as novel anti-hepatitis B virus agents. *Bioorganic & medicinal chemistry letters* **2014**, *24*, 2353-2359, doi:10.1016/j.bmcl.2014.03.060.

37. Gupta, K.K.; Taneja, S.C.; Dhar, K.L.; Atal, C.K. Flavonoids of Andrographis-Paniculata. *Phytochemistry* **1983**, *22*, 314-315, doi:Doi 10.1016/S0031-9422(00)80122-3.

38. Chen, L.X.; He, H.; Xia, G.Y.; Zhou, K.L.; Qiu, F. A new flavonoid from the aerial parts of Andrographis paniculata. *Nat Prod Res* **2014**, *28*, 138-143, doi:10.1080/14786419.2013.856907.

39. Lee, J.C.; Tseng, C.K.; Young, K.C.; Sun, H.Y.; Wang, S.W.; Chen, W.C.; Lin, C.K.; Wu, Y.H. Andrographolide exerts anti-hepatitis C virus activity by up-regulating haeme oxygenase-1 via the p38 MAPK/Nrf2 pathway in human hepatoma cells. *British journal of pharmacology* **2014**, *171*, 237-252, doi:10.1111/bph.12440.

40. Praveen, N. Polyphenol composition and antioxidant activity of Andrographis paniculata L. Nees. *Mapana Journal of Sciences* **2014**, *13*, 33-46.

41. Dua, V.K.; Qjha, V.P.; Roy, R.; Joshi, B.C.; Valecha, N.; Devi, C.U.; Bhatnagar, M.C.; Sharma, V.P.; Subbatao, S.K. Anti-malarial activity of some xanthones isolated from the roots of *Andrographis paniculata*. *Journal of Ethnopharmacology* **2004**, *95*, 247-251, doi:10.1016/j.jep.2004.07.008.

42. Dua, V.K.; Verma, G.; Dash, A.P. In Vitro Antiprotozoal Activity of Some Xanthones Isolated from the Roots of Andrographis paniculata. *Phytotherapy Research* **2009**, *23*, 126-128, doi:10.1002/ptr.2556.

43. Xu, C.; Chou, G.X.; Wang, C.H.; Wang, Z.T. Rare noriridoids from the roots of Andrographis paniculata. *Phytochemistry* **2012**, *77*, 275-279, doi:10.1016/j.phytochem.2011.12.020.
