# Supplementary material for: Andrographis paniculata (Burm. f.) Wall. ex Nees: An Updated Review of Phytochemistry, Antimicrobial Pharmacology, and Clinical Safety and Efficacy
Source: Life (Basel). 2021 Apr 16;11(4):348. doi: 10.3390/life11040348 (PMC8072717; doi:10.3390/life11040348)
Supplement: Supplementary file 1 [file life-11-00348-s001.zip › life-1140970/Supplementary Table S3.docx]

**Table S3.** Study characteristics and intervention outcomes of clinical trials of *Andrographis paniculata* for healthy volunteers and other health complications included familial Mediterranean fever, diabetes mellitus, hypertriglycemia, ulcerative colitis, fatigue and arthritis pain.

| Study ID;  Year;  Country | Study Design | Gender & Age | Recruitment(n)/ Analyzed(n) | Diagnosis | Study Medications | Daily Dosage (duration) | Active Ingredients | Salient Outcomes | Adverse Effects (cases) |
| --- | --- | --- | --- | --- | --- | --- | --- | --- | --- |
| Amaryan, *et al.* [1];  2003;  Armenia | R, DBPC | G: M&F A: 2-15 y | n = 29/24  CR = 82.76%  AP = 14/14  P = 10/10  BCS = NSD (p>0.05) | Familial Mediterranean Fever (FMF) | ImmunoGuard® | 4 tablets 3 xD (1 m) | Mainly AND, EE, Schisandrins, Glycyrrhizin | ImmunoGuard® showed significant efficacy to manage FMF patients. It was safe to treat FMF. | No side effects were observed. |
| Bertoglio, *et al.* [2];  2016;  Chile | R, DBPC | G: M&F A: 18-50 y | n = 24/21  CR = 87.5%  AP = 13/11  P = 11/10  BCS = NSD (p>0.05) | Fatigue | AP purified extract (170 mg) contains AND (85 mg/tablet) | 1 tablet 2 xD (12 m) | 85 mg/tablet AND | AP extract treatment showed a significant reduction of 44 % in the FSS score compared to placebo at 12 months, and this effect seemed to be time dependent. AP was safe and well tolerated, and no changes in clinical parameters were observed. | Mild and transient skin rash (1) |
| Suriyo, et al. [3];  2017;  Thailand | Open-label | G: M&F  A: | n = 20/20  CR = 100%  AP = 20/20 | Healthy volunteer  (To investigate the physiological effect of APE used for common cold and URTI) | APE crude power (350 mg/capsule) | 4 capsules 3 xD (3 d) | 8.16 mg AND, 0.90 mg NAND, 0.96 mg DAND, and 1.35 mg DDHAND per capsule | Multiple-dose treatment of APE conserves normal haematological profiles, blood chemistry parameters, blood coagulation parameters, electrocardiogram, or urine analysis parameters in healthy volunteers. | No serious adverse events reported in subjects during the treatment period. |
| Mkrtchyan, *et al.* [4];  2005;  Armenia | RPG | G: M&F A: 18-35 y | n = 12 (healthy volunteers take part twice)  G1 = 5/5  G2 = 5/5  G3 = 6/6  G4 = 5/5  G5 = 5/5  BCS = NSD (p>0.05) | Healthy volunteer (To determine the effect of APE on the semen quality and fertility) | M1: 6 mL APE (526 mg, 60 mg AND)  M2: 6 mL APE (1052 mg, 120 mg AND)  M2: 9 mL APE (1578 mg, 180 mg AND)  M3: 40 mL ginseng (3 g, 85.7 mg ginsenoside)  M4: 5 tablets valerian (2.5-4 g) | G1 = 2 mL (M1) 3 xD (13 d)  G2 = 2 mL (M2) 3 xD (13 d)  G3 = 3 mL (M2) 3 xD (13 d)  G4 = 20 mL (M3) 2 xD (13 d)  G5 = 2 tablets and 3 tablets daily (13 d) | AND, ginsenoside, and valerian | Use of APE is safe. Three times higher daily recommended dose of APE (equivalent to 180 mg AND daily) does not have adverse effect on semen quality. Extracts of *P. ginseng* and VO were also shown to have no effect on human male sterility at levels corresponding to approximately three human daily doses. | No serious adverse events or side effects were recorded. |
| Chen, *et al.* [5];  2012;  China | R3WC | G: M&F A: 18-40 y | n = 9/9  CR = 100%  G1 = 3/3  G2 = 3/3  G3 = 3/3  BCS = NSD (p>0.05) | Healthy volunteer (To investigate pharmacokinetics and tolerance ability) | APE: DAS (80 mg/vial) | G1: IV 80 mg daily | DAS | DAS injection is safe and well-tolerated over the dose range (single doses of DAS injection up to 320 mg) of IV infusion in young, healthy male and female subjects. DAS is rapidly cleared from the blood, and most of the drug was excreted after transformation to metabolites. | Mild stomachache (1). No other serious or unexpected adverse events were observed. |
| Phunikhom, *et al.* [6];  2015;  Thailand | R, RNB | G: M&F A: 20-65 y | n = 60/54  CR = 90%  LDG (AP) = 20/18  HDG (AP) = 20/17  Ctr = 20/19  BCS = NSD (p>0.05) | Hypertriglyceridemia | AP: APE (AND 24 mg/capsule)  Ctr.: Gemfibrozil (300 mg/capsule) | LDG: 1 capsule 3 xD (8 w); HDG: 5 capsules 2, 1, 2 daily (8 w) Ctr. 1 capsule daily (8 w) | 24 mg/capsule AND | APE decreased the level of serum triglyceride significantly (p=0.0442) without any effect on serum HDL-C, liver enzymes and creatine kinase. AP extract treatment was as tolerable as gemfibrozil treatment. | Nausea (5): LDG (2), HDG (3) |
| Tang, *et al.* [7];  2011;  China | RDB-PG | G: M&F A: 18-65 y | n = 120/108  CR = 90%  AP = 60/53  Ctr = 60/55  BCS = NSD (p>0.05) | Mild-to-moderate active ulcerative colitis | AP: APE (400 mg/tablet)  Ctr.: mesalamine SR granules (1500 mg/tablet) | 1200 mg APE daily and 1500 mg mesalamine daily (8 w) | AND | APE has efficacy similar to slow release mesalazine and was well tolerated in patients with mildly to moderately active Ulcerative colitis. | APE: Aphthous ulcer (1), WBC decrease (1), Abdominal pain (1), Blood in the stool (1), Fever (1), Elevated glucose (1), Rash (1); Mesalamine:  Blood in the urine (2), Elevated CRP (1), WBC decrease (1), Blood in the urine (2), Fever (1), WBC decrease (1), Abdominal pain (1), Dry mouth (1), Oedema lower extremity (1) Cough (2), Diarrhoea (2), Dizziness and nausea (1), WBC elevated in urine (1) and other (increased platelets, total bilirubin, joint pain) (3) |
| Sandborn, et al. [8];  2013;  The United States,  Canada, Germany, Romania, and Ukraine | R, DBPC | G: M&F A: at least 18 y | n = 244/180  CR = 73.77%  LDG (AP) = 75/57  HDG (AP) = 74/59  P = 75/64  BCS = NSD (p>0.05) | Mild-to-moderate ulcerative colitis | AP: AP ethanol extract. | LDG: 400 mg 3 xD (8 w); HDG: 600 mg 3 xD (8 w) |  | Clinical response was significantly higher in the AP group than those in placebo p=0.0183. The best efficacy was observed with the HDG. | The serious adverse event observed (2). |
| Hancke, *et al.* [9];  2019;  India | R, DBPC | G: M&F A: 40-70 y | n = 108/103  CR = 95.37%  LDG (AP) = 37/33  HDG (AP) = 35/35  P = 36/35  BCS = NSD (p>0.05) | Pain in osteoarthritis | LDG: Each gelatin capsule contains 150 mg APE purified (total 75 mg AND);  HDG: Each gelatin capsule contains 300 mg APE purified (total 150 mg AND) | 1 capsule 2 xD (12 w) | 75 or 150 mg AND | A significant reduction in pain found in ParActin® group compare to placebo | Gastrointestinal symptoms (8): acidity (4-LDG), (2-P), constipation (1-LDG), and oral ulcers (1-LDG). Alanine aminotransferase (3): HDG(1) and LDG (2) |
| Burgos, *et al.* [10];  2009;  Chile | R, DBPC | G: F  A: 18-70 y | n = 60/58  CR = 96.67%  AP = 30/30  P = 30/28  BCS = NSD (p>0.05) | Rheumatoid arthritis | AP: Standardized dried APE contains AND (30 mg/tablet) | 1 tablet 3 xD (14 w) | 30 mg/tablet AND | A reduction of pain and swelling and other clinical parameters in AP treated group. However, there was NSD between TrG and placebo. | Pruritus (1). Nausea, diarrhoea, and stomach discomfort was equally reported in both groups. |
| Widjajakusuma, *et al.*  [11];  2019;  Indonesia | R, DBPC | G: M&F A: ≥30 y | n = 66/54  CR = 81.82%  AP = 32/27  P = 34/27  BCS = NSD (p>0.05) | Type 2 diabetes mellitus | AP: Metformin (500 mg/tablet) + Mixture of APE and SP extract (450 mg/tablet);  P: Metformin only | 1 tablet 2 xD (8 w) | Glycosides, terpenoids, alkaloids, flavonoids, saponins, and tannins. | Improved the decrease of fasting and postprandial glucose and significantly lowered BMI compare to control group. AP treatment is a promising therapeutic option for liver and kidney as well. | No adverse event information reported. |

**Table S3.** Registered clinical trials database of *A. paniculata* on <https://clinicaltrials.gov/>.

| **NCT**  **number** | **Year** | **Phase** | **Status** | **Population** | **Conditions** | **Interventions** | **Study type** | **Study design** | **Outcome measures** | **Sponsor/collaborator** | **Location** |
| --- | --- | --- | --- | --- | --- | --- | --- | --- | --- | --- | --- |
| NCT04196075 | 2018 | Phase 3 | R | E: 30  A: 18 y  Older Adult  Sex: All | Squamous Cell Carcinoma of Esophagus | AP | Invl. | •Allocation: N/A  •Intervention Model: Single Group Assignment  •Masking: None (Open Label)  •Primary Purpose: Supportive Care | •Symptomatic relief and quality of life after AP  •Difficulty to swallow  •Survival  •Adverse events  •AP side effects | •Chinese University of Hong Kong | •Department of Surgery, Faculty of Medicine, The Chinese University of Hong Kong, Hong Kong, Outside Of US & Canada, China |
| NCT04161404 | 2019 | Phase 1 | R | E: 18  A: 18-45 y  Sex: M | •Pharmacokinetics  •Molecular Mechanisms of Pharmacological Action | • AP 1000 mg capsules  • AP 2000 mg  • Metformin (Glucophage) 1000 mg | Invl. | •Allocation: Randomized  •Intervention Model: Crossover Assignment  •Masking: None (Open Label)  •Primary Purpose: Other | •Area under the plasma concentration-time curve (AUC) of Andrographis paniculata  •Cmax of Andrographis paniculata  •Tmax of Andrographis paniculata  •Area under the plasma concentration-time curve (AUC) of Metformin  •Cmax of Metformin  •Tmax of Metformin  •Metabolic pathway of Andrographis paniculata  •Metabolic pathway of Metformin  •Adverse drug reaction | •University of Malaya  •Ministry of Health, Malaysia | •Clinical Investigation Centre, University Malaya Medical Centre, Kuala Lumpur, Wilayah Persekutuan Kuala Lumpur, Malaysia |
| NCT03455049 | 2017 | NA | C | E:73  A: 18-60 y  Sex: All | •Increased Insulin | • AP Extract | Invl. | •Allocation: Randomized  •Intervention Model: Crossover Assignment  •Masking: Double (Participant, Investigator)  •Primary Purpose: Treatment | GLP-1  •Fasting Insulin  •2h-OGTT Insulin  •HOMA-IR  •Fasting plasma glucose level  •2h-OGTT plasma glucose level  •Dypeptidil Peptidase 4 (DPP-4) Enzyme  •Glycated Albumin | •Indonesia University | Department of Surgery, Faculty of Medicine, The Chinese University of Hong Kong, Hong Kong, Outside Of US & Canada, China |
| NCT04463875 | 2018 |  | C | E: 113  Age:  Child  Adult  OA  Sex: All | Migraine | Dietary Supplement: magnesium, vitamin B2, fever few, AP and coenzyme Q10 | Obsl. | •Observational Model: Case-Only  •Time Perspective: Prospective | •Change in monthly migraine days  •Mean intensity of migraine  •days with use of acute migraine medications  •Migraine Disability Assessment questionnaire (MIDAS)  •Headache Impact Test-6 (HIT-6)  •Migraine Therapy Assessment questionnaire (MTAQ) | Corfu Headache Clinic | Corfu HC, Corfu, Greece |
| NCT02280876 | 2012 | Phase 1  Phase 2 | C | E: 30  A: 18-55 y  Sex: All | Multiple Sclerosis, Relapsing Remitting | • AP p/st extract  • Excipients | Invl. | •Allocation: Randomized  •Intervention Model: Parallel Assignment  •Masking: Triple (Participant, Care Provider, Investigator)  •Primary Purpose: Treatment | •Clinical inflammatory (stamina) and disability score parameters (sensorial, neurosensitive, neuromotor and cognitive function) in patients with RRMS, treated with ApE and placebo administered over beta interferon.  •Safety, tolerability and efficacy of ApE and placebo administered over beta interferon in RRMS treated patients (adverse symptoms, general clinical laboratory and comparative statistical parameter | •Universidad Austral de Chile  •Comisión Nacional de Investigación Científica y Tecnológica  •University of Chile | Hospital Regional , Valdivia,Los Rios,Chile |
| NCT03780621 | 2019 | Phase 1 | C | E: 16  A: 60-75 y  Sex: All | Cognitive Impairment, Mild | Dietary Supplement: AP and Withania  •Dietary Supplement: Placebo | Invl. | •Allocation: Randomized  •Intervention Model: Crossover Assignment  •Masking: Triple (Participant, Care Provider, Investigator)  •Primary Purpose: Treatment | Change over time in neural electrical activity of the brain as measured by quantitative-topographic EEG for herbal treatment group versus placebo | •EuroPharma, Inc. | •Clinical Labors of NeuroCode AG, Wetzlar, Germany |
| NCT03190044 | 2017 | NA | R | E: 82  A: 18-65 y  Sex: All | •Migraine Disorders | •Dietary Supplement: PACR  •Dietary Supplement: Placebo | Invl. | •Allocation: Randomized  •Intervention Model: Parallel Assignment  •Masking: Single (Care Provider)  •Primary Purpose: Prevention | •Migraine improvement in terms of responder rate  •Migraine improvement in terms of frequencies | •University of Roma La Sapienza  •Gianluca Coppola  •Francesco Pierelli | •Policlinico Umberto I, Rome, Italy |
| NCT02003651 | 2013 | NA | C | E: 40  A: 18-55 y  Sex: F | •Acute Respiratory Infections | •Dietary Supplement: Quick Defense  •Dietary Supplement: Placebo | Invl. | •Allocation: Randomized  •Intervention Model: Parallel Assignment  •Masking: Double (Participant, Investigator)  •Primary Purpose: Prevention | Common cold symptoms | •Appalachian State University  •Gaia Herbs Inc | ASU-NCRC Human Performance Lab, Kannapolis, North Carolina, United States |
| NCT02539277 | 2014 | Phase 4 | U | E: 600  A: 18-70 y  Sex: All | •Acute Upper Respiratory Infection | •Drug: Jinyebaidu granule  •Drug: Fufangshuanghua granule  •Drug: Jinyebaidu granule placebo  •Drug: Fufangshuanghua granule placebo | Invl. | •Allocation: Randomized  •Intervention Model: Parallel Assignment  •Masking: Quadruple (Participant, Care Provider, Investigator, Outcomes Assessor)  •Primary Purpose: Treatment | •The time to defervescence  •Change of symptoms score  •Change of signs score  •Adverse events  •Change of routine blood test  •Change of urine routine  •Change of liver function  •Change of renal function  •Incidence of ECG abnormalities | •China Academy of Chinese Medical Sciences  •First Affiliated Hospital of Heilongjiang Chinese Medicine University  •Guang'anmen Hospital of China Academy of Chinese Medical Sciences  •Beijing Hospital of Traditional Chinese Medicine  •Changchun University of Chinese Medicine  •Liaoning University of Traditional Chinese Medicine  •Shandong University of Traditional Chinese Medicine  •Guangdong Provincial Hospital of Traditional Chinese Medicine | •Guang'anmen Hospital, Beijing, Beijing, China |
| NCT00749645 | 2006 | Phase 2 | C | E: 60  A: 18-70 y  Sex: All | Arthritis,  Rheumatoid | • FANG(30)  • Placebo | Invl. | • Allocation: Randomized  • Intervention Model: Parallel  Assignment  • Masking: Quadruple  (Participant, Care Provider,  Investigator, Outcomes  Assessor)  • Primary Purpose:  Treatment | •Primary: Day1, end of  week2, then every 4  weeks for 105 days, the  following will be measured:  1.Inflamed and painful  joints count. 2. Intensity of  pain by patient on Visual  Analogue Scale(VAS).  3. Overall improvement  by HAQ and SF 36  (enclosed).  •Secondary:Day1, end  of week2, then every 4  weeks for 3 mo, it will  be measured: Morning  stiffness duration.  •Symptoms relief by patient  and researcher.  •Paracetamol used as  rescue medicine for pain.  •Tolerability by patient and  researcher. | •Juan C.  Bertoglio, MD  •Fondo Nacional  de Desarrollo  Científico y  Tecnológico,  Chile  •Universidad  Austral de Chile | •HOSPITAL REGIONAL de  OSORNO, Osorno, Los Lagos,  Chile  •Hospital Clínico Regional,  Valdivia, Los Ríos, Chile |

C: Completed, R: Recruiting, U: Unknown, F: Female, M: Male, A: Age, y: Year

**Reference**

1. Amaryan, G.; Astvatsatryan, V.; Gabrielyan, E.; Panossian, A.; Panosyan, V.; Wikman, G. Double-blind, placebo-controlled, randomized, pilot clinical trial of ImmunoGuard--a standardized fixed combination of Andrographis paniculata Nees, with Eleutherococcus senticosus Maxim, Schizandra chinensis Bail. and Glycyrrhiza glabra L. extracts in patients with Familial Mediterranean Fever. *Phytomedicine* **2003**, *10*, 271-285, doi:10.1078/094471103322004767.

2. Bertoglio, J.C.; Baumgartner, M.; Palma, R.; Ciampi, E.; Carcamo, C.; Caceres, D.D.; Acosta-Jamett, G.; Hancke, J.L.; Burgos, R.A. Andrographis paniculata decreases fatigue in patients with relapsing-remitting multiple sclerosis: a 12-month double-blind placebo-controlled pilot study. *BMC Neurol* **2016**, *16*, 77, doi:10.1186/s12883-016-0595-2.

3. Suriyo, T.; Pholphana, N.; Ungtrakul, T.; Rangkadilok, N.; Panomvana, D.; Thiantanawat, A.; Pongpun, W.; Satayavivad, J. Clinical Parameters following Multiple Oral Dose Administration of a Standardized Andrographis paniculata Capsule in Healthy Thai Subjects. *Planta Med* **2017**, *83*, 778-789, doi:10.1055/s-0043-104382.

4. Mkrtchyan, A.; Panosyan, V.; Panossian, A.; Wikman, G.; Wagner, H. A phase I clinical study of Andrographis paniculata fixed combination Kan Jang versus ginseng and valerian on the semen quality of healthy male subjects. *Phytomedicine* **2005**, *12*, 403-409, doi:10.1016/j.phymed.2004.10.004.

5. Chen, Q.; Liu, Y.; Liu, Y.M.; Liu, G.Y.; Zhang, M.Q.; Jia, J.Y.; Lu, C.; Yu, C. Pharmacokinetics and tolerance of dehydroandrographolide succinate injection after intravenous administration in healthy Chinese volunteers. *Acta Pharmacol Sin* **2012**, *33*, 1332-1336, doi:10.1038/aps.2012.79.

6. Phunikhom, K.; Khampitak, K.; Aromdee, C.; Arkaravichien, T.; Sattayasai, J. Effect of Andrographis paniculata Extract on Triglyceride Levels of the Patients with Hypertriglyceridemia: A Randomized Controlled Trial. *J Med Assoc Thai* **2015**, *98 Suppl 6*, S41-47.

7. Tang, T.; Targan, S.R.; Li, Z.S.; Xu, C.; Byers, V.S.; Sandborn, W.J. Randomised clinical trial: herbal extract HMPL-004 in active ulcerative colitis - a double-blind comparison with sustained release mesalazine. *Alimentary pharmacology & therapeutics* **2011**, *33*, 194-202, doi:10.1111/j.1365-2036.2010.04515.x.

8. Sandborn, W.J.; Targan, S.R.; Byers, V.S.; Rutty, D.A.; Mu, H.; Zhang, X.; Tang, T. Andrographis paniculata extract (HMPL-004) for active ulcerative colitis. *Am J Gastroenterol* **2013**, *108*, 90-98, doi:10.1038/ajg.2012.340.

9. Hancke, J.L.; Srivastav, S.; Caceres, D.D.; Burgos, R.A. A double-blind, randomized, placebo-controlled study to assess the efficacy of Andrographis paniculata standardized extract (ParActin(R)) on pain reduction in subjects with knee osteoarthritis. *Phytother Res* **2019**, *33*, 1469-1479, doi:10.1002/ptr.6339.

10. Burgos, R.A.; Hancke, J.L.; Bertoglio, J.C.; Aguirre, V.; Arriagada, S.; Calvo, M.; Caceres, D.D. Efficacy of an Andrographis paniculata composition for the relief of rheumatoid arthritis symptoms: a prospective randomized placebo-controlled trial. *Clin Rheumatol* **2009**, *28*, 931-946, doi:10.1007/s10067-009-1180-5.

11. Widjajakusuma, E.C.; Jonosewojo, A.; Hendriati, L.; Wijaya, S.; Ferawati; Surjadhana, A.; Sastrowardoyo, W.; Monita, N.; Muna, N.M.; Fajarwati, R.P., et al. Phytochemical screening and preliminary clinical trials of the aqueous extract mixture of Andrographis paniculata (Burm. f.) Wall. ex Nees and Syzygium polyanthum (Wight.) Walp leaves in metformin treated patients with type 2 diabetes. *Phytomedicine* **2019**, *55*, 137-147, doi:10.1016/j.phymed.2018.07.002.
